# Hydrogel‐Coated Foam Evaporator Achieving Four‐In‐One Benefits for Efficient and Stable Solar‐Driven Water Purification

**DOI:** 10.1002/advs.202517280

**Published:** 2025-11-12

**Authors:** Lele Li, Yanhong Dong, Shu Liu, Xingmin Du, Diandian Yu, Jie Wen, Chenxi Wang, Qian Sun, Bing Liu, Tao Wu, Jiahui Yu

**Affiliations:** ^1^ Medical Science and Technology Innovation Center Shandong First Medical University & Shandong Academy of Medical Sciences Jinan 250117 China; ^2^ Department of Chemical and Environmental Engineering The University of Nottingham Ningbo China Ningbo 315100 China; ^3^ Nottingham Ningbo China Beacons of Excellence Research and Innovation Institute University of Nottingham Ningbo China Ningbo 315100 China

**Keywords:** four benefits in one approach, hydrogel‐coated foam evaporator, solar‐driven evaporator, sustainable low‐carbon technology, universal strategy

## Abstract

Interfacial solar evaporation has emerged as an innovative water purification strategy due to its environmental friendliness, high efficiency, adaptability to various water sources. This study introduces a universal strategy for foam‐based solar evaporators, leveraging unique advantages of hydrogel materials to effectively reduce evaporation enthalpy. At the same time, it addresses challenges such as the need for pore formation in hydrogels, water‐induced swelling, and the detachment of photothermal materials, achieving “four benefits in one approach.” The fabrication process takes only 40 min and features an extremely low carbon footprint (0.00462 kg CO_2_ eq kg^−1^). After modification, the evaporation performance of different foam‐based evaporators improved by 15%62%, with the melamine foam‐based evaporator showing the most notable enhancement (62%). Using melamine foam as the supporting material, a PVA‐melamine foam composite hydrogel evaporator (FCH) is successfully fabricated via the hydrogel‐based improvement technique. FCH evaporator achieved an exceptional evaporation rate of 7.93 ± 0.37 kg m^−2^ h^−1^ under 1 sun irradiation and maintained excellent stability during 30‐day continuous seawater test. Owing to the superior stability of the hydrogel thin layer, FCH evaporator retained high evaporation performance even under extreme conditions. This study provides new insights and practical solutions for advancing sustainable water purification technologies.

## Graphical abstract

This study introduces a universal strategy for foam‐based solar evaporators, leveraging the unique advantages of hydrogel materials to effectively reduce evaporation enthalpy. At the same time, it addresses challenges such as the need for pore formation in hydrogels, water‐induced swelling, and the detachment of photothermal materials, achieving “four benefits in one approach.”

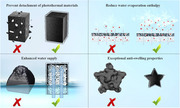

## 1 Introduction

In the context of global efforts to combat climate change and achieve carbon neutrality, the transition to low‐carbon energy has become an urgent priority.^[^ ^1^ ^]^ Traditional seawater desalination technologies, such as membrane separation techniques^[^ ^2^, ^3^ ^]^ and thermal distillation methods,^[^ ^4^ ^]^ typically rely on fossil fuels, resulting in high energy consumption, high operational costs, and the discharge of large volumes of concentrated brine, imposing significant environmental burdens. Recently, solar‐driven desalination has emerged as an innovative and sustainable alternative,^[^ ^5^, ^6^, ^7^, ^8^, ^9^, ^10^, ^11^ ^]^ offering the potential to significantly reduce energy consumption, mitigate environmental impacts, and achieve “zero emissions”, thereby contributing to global carbon neutrality goals.

In the field of solar desalination, hydrogel materials have garnered considerable attention due to their ability to effectively reduce the evaporation enthalpy of water.^[^ ^12^ ^]^ Additionally, hydrogels exhibit fast gelation and simple preparation processes, making them ideal candidates for the rapid fabrication of evaporators.^[^ ^13^, ^14^ ^]^ However, conventional non‐porous hydrogels suffer from slow water uptake after dehydration, often requiring several hours to recover, which severely limits the continuous water supply during evaporation.^[^ ^15^ ^]^ To address this limitation, researchers have increasingly focused on porous hydrogels,^[^ ^16^ ^]^ as their porous structures significantly enhance water absorption rates, positioning them as a key focus in evaporator design.

Common strategies for introducing porosity in hydrogels include repeated freeze‐thaw cycles,^[^ ^17^ ^]^ freeze‐drying,^[^ ^18^, ^19^ ^]^ and sacrificial template methods.^[^ ^20^ ^]^ Despite their effectiveness, these techniques have notable limitations: freeze‐thaw cycles are energy‐intensive and involve laborious processes under low temperatures; freeze‐drying requires prolonged treatment in vacuum conditions, making it time‐consuming and energy‐demanding;^[^ ^21^ ^]^ and sacrificial template methods often involve toxic solvents to dissolve templates,^[^ ^22^ ^]^ which can pose environmental risks.^[^ ^23^, ^24^, ^25^ ^]^ Therefore, developing a low‐energy, low‐carbon approach to address water supply issues in hydrogels while maintaining their superior evaporation performance is of critical importance for achieving rapid, efficient, and environmentally friendly evaporator fabrication.

In recent years, 3D foam‐based evaporators have attracted widespread attention due to their excellent thermal insulation properties, superior water supply capabilities, and simple, adaptable structural designs.^[^ ^26^, ^27^, ^28^, ^29^, ^30^, ^31^ ^]^ The unique characteristics of foam‐based structures allow them to retain heat effectively and provide sufficient water supply, thereby enhancing evaporation efficiency. However, current foam‐based evaporators face significant challenges, such as the instability of photothermal materials and the high evaporation enthalpy of water.^[^ ^30^, ^32^ ^]^ If these issues can be addressed through simple design and optimization—ensuring the stability of photothermal materials while effectively reducing water evaporation enthalpy—the improvement method for foam‐based evaporators could further drive advancements in 3D foam‐based evaporator technologies.

**Scheme 1:**
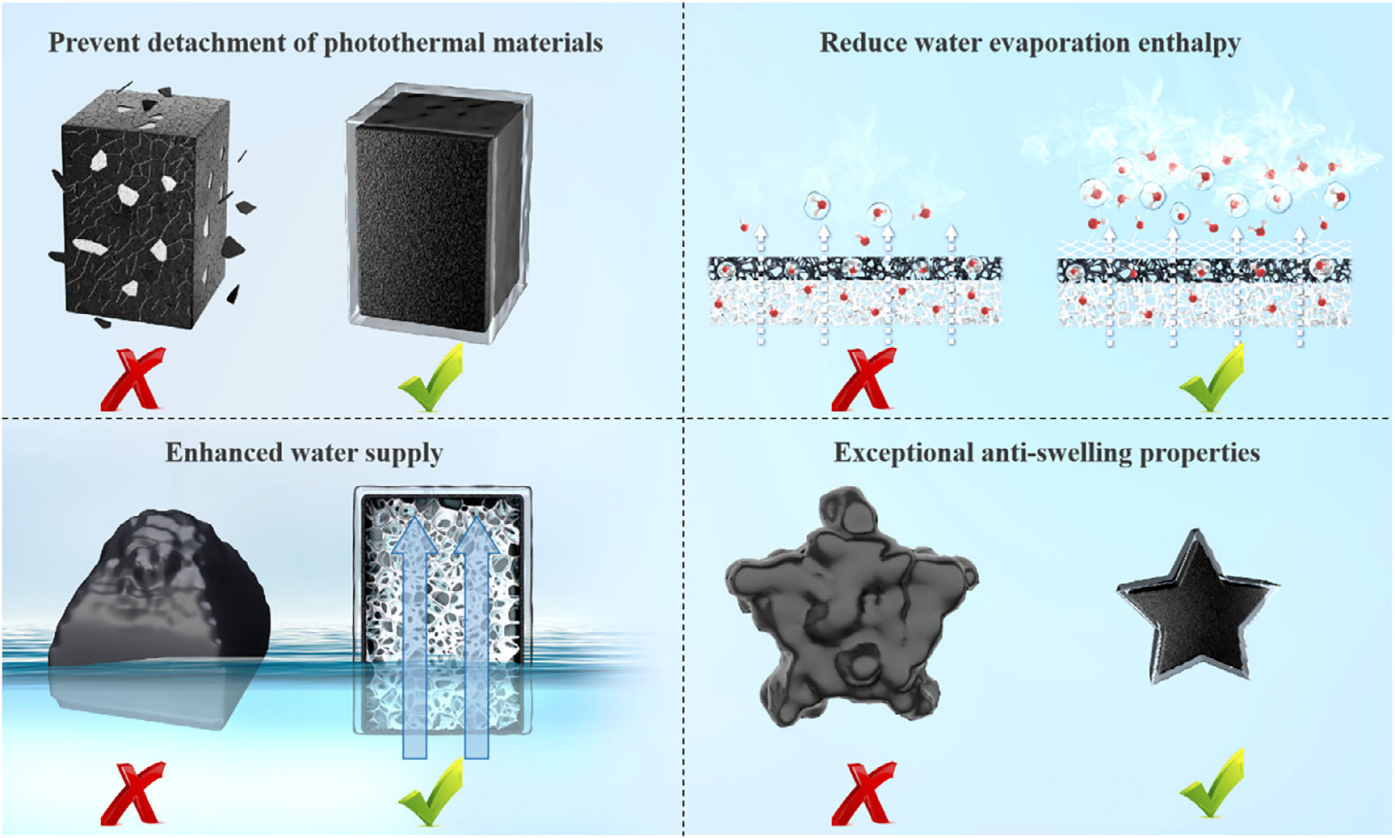
"Four benefits in one approach" of the FCH.

This study demonstrates a precise thickness modulation strategy for hydrogel coatings on foam‐based evaporators, which optimally balances the trade‐off between the reduced water evaporation enthalpy enabled by hydrogel materials and their inherent water supply limitations. This approach achieves remarkable evaporation enhancement while maintaining a facile fabrication process. As illustrated in **Scheme** **1**, compared to pristine foam evaporators, our designed hydrogel‐coated foam evaporator not only effectively prevents photothermal material detachment but also significantly reduces water evaporation enthalpy through the hydrogel thin layer. In contrast to pure hydrogel evaporators, this architecture exhibits superior continuous water supply capability, eliminating the surface wrinkling issue caused by water deficiency in conventional hydrogels, while maintaining excellent anti‐swelling properties and structural stability across various aqueous environments.

**Scheme 2:**
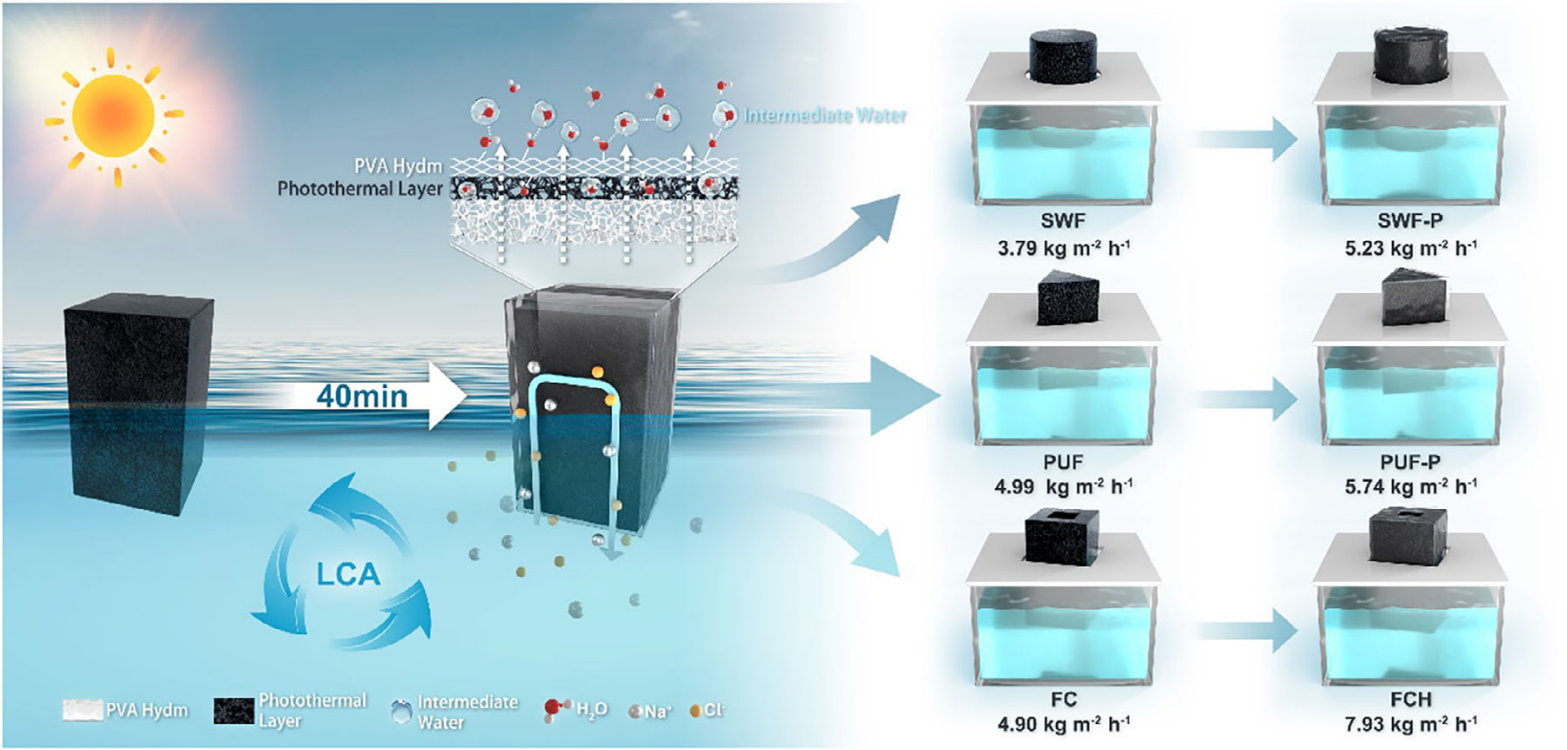
Improved methods for universal foam‐based evaporators and application in SWF, PUF, and FC‐based evaporators.

More significantly, this universal modification method demonstrates substantial efficacy across diverse foam substrates, achieving 15%–62% performance enhancement in solar desalination (**Scheme** **2**). The entire modification process requires merely 40 min with a minimal carbon footprint (0.00462 kg CO_2_ eq kg^−1^).^[^ ^33^ ^]^ Particularly noteworthy is the FCH (PVA‐melamine foam composite hydrogel evaporator) fabricated using this technique, which achieves an exceptional evaporation rate of 7.93 ± 0.37 kg m^−2^ h^−1^ under 1 sun irradiation while demonstrating outstanding salt rejection in 20 wt.% high‐salinity brine. These results unequivocally confirm the system's tremendous potential for practical applications

## 2 Results and Discussion

### 2.1 In Situ PVA Thin Layer Modification

The universal foam‐based evaporator modification method proposed in this study involves the in situ formation of a polyvinyl alcohol hydrogel thin layer (PVA hydm), as shown in Scheme 2. The hydrogel thin layer is formed on the outer surface of the foam‐based evaporator without disrupting the internal pore structure of the foam, thus preserving its excellent water supply capability. On the other hand, the hydrogel thin layer formed on the outer surface leverages the unique properties of the hydrogel to lower the evaporation enthalpy of water molecules, thereby significantly improving evaporation efficiency. Moreover, the hydrogel thin layer effectively secures the photothermal material, preventing its detachment during use and further improving the stability and long‐term performance of the evaporator.

As shown on the right side of Scheme 2, seaweed foam (SWF), polyurethane foam (PUF) and melamine foam‐based composite with photothermal material (FC) exhibit lower evaporation rates, after the improvement with this method, meaning to coat foam substrates with thin layer of PVA hydrogel, different types of foam‐based evaporators, including seaweed foam‐PVA hydm (SWF‐P), polyurethane foam‐PVA hydm (PUF‐P) and melamine foam‐based composite with PVA hydm containing photothermal material (FCH) evaporators, all achieved significant performance improvements ranging from 15% to 62% (Figure S1, Supporting Information). This indicates that the proposed method has strong versatility and can effectively enhance the evaporation performance of various foam‐based materials.

It is worth noting that this simple hydrogel modification method can be completed within 40 minutes without requiring any additional energy input. According to the life cycle assessment (LCA) analysis in **Table** **1**, the carbon emissions of this process are as low as 0.00462 kg CO_2_ eq kg^−1^. Compared to the pore formation of hydrogels via foaming freeze‐drying^[^ ^19^ ^]^ or repeated freeze‐thaw cycles,^[^ ^17^ ^]^ this hydrogel modification method achieves a reduction in carbon emissions by 97.03% and 98.73%, respectively, demonstrating significant potential for carbon reduction and efficiency enhancement.

**Table 1:** All potential carbon emissions associated with different hydrogel preparation methods.

	Material [kg CO_2_ eq kg^−1^]	Energy [kg CO_2_ eq kg^−1^]	Overall [kg CO_2_ eq kg^−1^]
Foaming method	0.00681	0.14878	0.15560
Freeze‐thaw method	0.00910	0.35720	0.36631
**This method**	0.00297	0.00164	0.00462

### 2.2 Mechanism Analysis of the FCH Evaporator for Seawater Desalination

**Figure 1:**
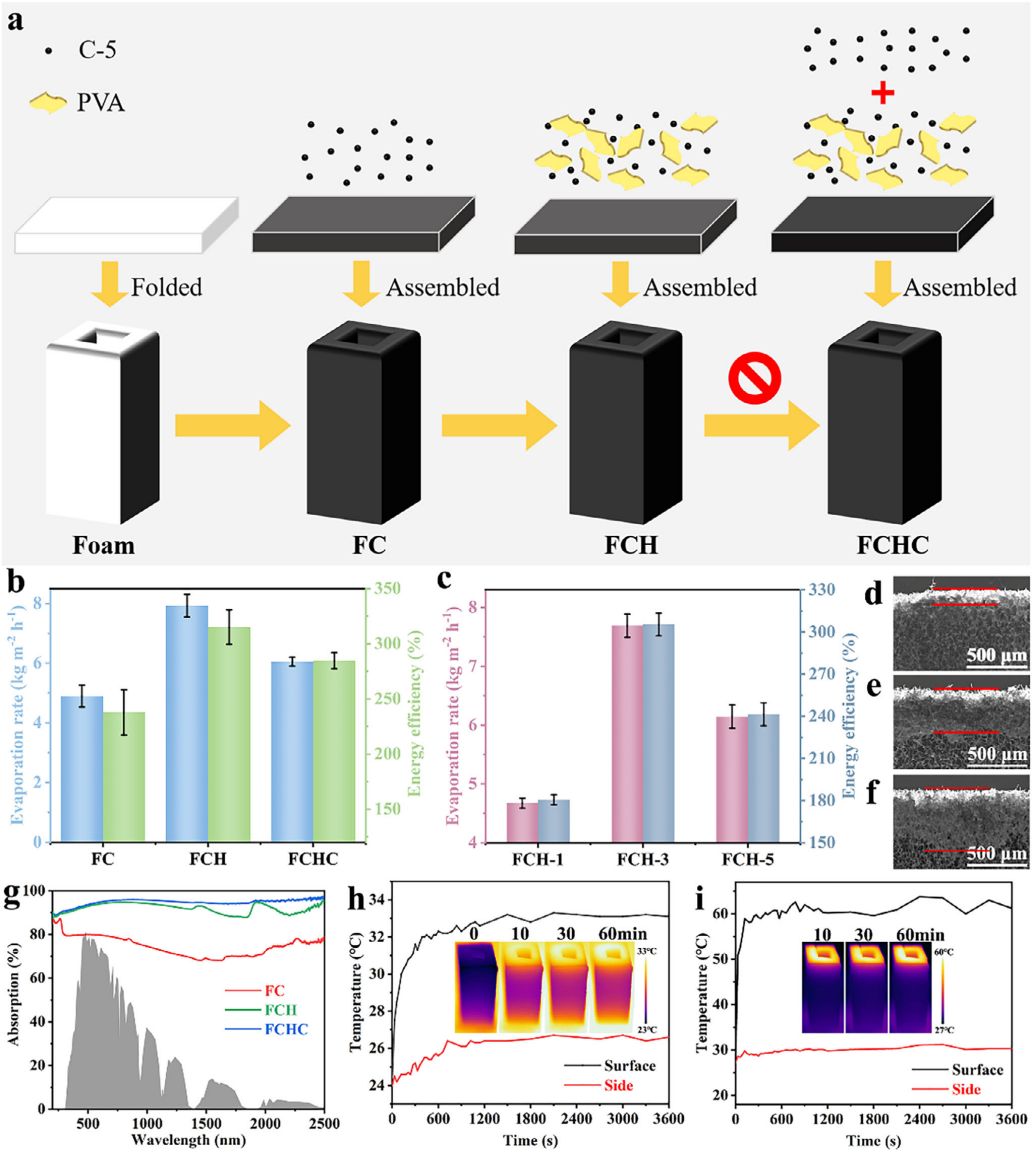
a) Preparation of FC, FCH, and FCHC. b) Evaporation rate and energy efficiency of FC, FCH, FCHC and c) FCH‐2, FCH‐3, FCH‐4. SEM of d) FCH‐2, e) FCH‐3 and f) FCH‐4. g) Absorption spectra of FC, FCH, and FCHC. h) Wet and i) dry FCH top and side temperature curves over time, an infrared image showing FCH temperature distribution for part of the period.

**Figure** **1**a illustrates the preparation process of series melamine foam‐based evaporators, where the primary distinction between FC and FCH lies in the absence or presence of a PVA hydm. The FCHC (foam‐based composite with PVA hydm and additional photothermal coating) evaporator is further modified by an additional spray coating of photothermal material on the FCH evaporator. The evaporation rates of these different evaporators are presented in Figure 1b. The FC evaporator demonstrates excellent water evaporation performance, with an evaporation rate and energy efficiency of 4.90 ± 0.36 kg m^−2^ h^−1^ and 237 ± 20.53% (calculation without environmental heat exchange). Incorporating environmental heat exchange, the recalculated energy efficiency for each evaporator in Table S1 (Supporting Information). The FCH evaporator, enhanced by the in situ formation of the PVA hydm, shows significant performance improvements, achieving an evaporation rate of 7.93 ± 0.37 kg m^−2^ h^−1^ and an energy efficiency of 315 ± 15.67%. This enhancement can be attributed to the intermediate water (IW) within the hydrogel thin layer, which effectively reduces the evaporation enthalpy of water molecules.^[^ ^34^ ^]^ The liquid water transitions through the IW, directly vaporizing into water vapor, thereby achieving a higher evaporation rate.

In contrast, the FCHC evaporator exhibits a slightly lower evaporation rate and energy efficiency of 6.05±0.14 kg m^−2^ h^−1^ and 284±7.25%, respectively. This reduction is due to the additional photothermal layer, which imposes certain constraints on the IW vaporization process, slowing the escape of water vapor and ultimately leading to a decrease in evaporation rate and energy efficiency compared to the FCH evaporator. Figure 1c compares the water evaporation performance of FCH evaporator with different hydrogel spray thicknesses. Under identical illumination condition, the evaporation rates of FCH‐1, FCH‐3, and FCH‐5 are 4.67 ± 0.08 , 7.69 ± 0.19 , and 6.14 ± 0.19 kg m^−2^ h^−1^, respectively. SEM images in Figure 1d–f show that the hydrogel thin‐layer thicknesses of FCH‐1, FCH‐3, and FCH‐5 are 150, 370, and 540 µm, respectively, indicating a direct correlation between hydrogel thickness and evaporation rate. For FCH‐1, the limited IW content results in minimal enhancement of the evaporation process. In contrast, the thicker hydrogel layer in FCH‐5 suggests the restriction for effective water supply (Figure S2, Supporting Information), the extended diffusion pathway FCH‐5 impedes the outward migration of IW and its timely replenishment at the evaporation interface, ultimately leading to a reduction in both the effective IW/FW ratio and evaporation efficiency. Therefore, a hydrogel thickness of ≈ 370 µm is optimal for improving evaporation performance. Additionally, a comparison of the evaporation performance of coating photothermal material which was carbonized in N_2_ atmosphere at 500 °C for 2 h (C‐5) with other photothermal materials highlights the significant advantages of C‐5 as a photothermal material (Figure S3, Supporting Information). Consequently, C‐5 was selected as the photothermal material for the preparation of FCH evaporators for further investigation.

Figure 1g and Equation S8 (Supporting Information) illustrate the light absorption capabilities of the evaporators across the 200–2500 nm wavelength range. The light absorption rates for FC, FCH, and FCHC are 74.65%, 91.97%, and 94.68%, respectively. Compared to FC, the addition of a hydrogel thin layer on the surface of FCH significantly enhances its light absorption capacity. For FCHC, the additional photothermal material layer on top of the hydrogel further increases light absorption by introducing surface roughness, which induces multiple light reflections. However, despite its higher light absorption, FCHC exhibits inferior water evaporation performance compared to FCH. The reason might be the additional layer of photothermal coating does not effectively reduce the enthalpy of evaporation, and even increases it somehow, hindering the formation of IW. Therefore, we speculate that the evaporation enthalpy can be effectively reduced only when the hydrogel thin layer is kept in the outermost layer. Meanwhile, the photothermal coating is in the outermost layer in FCHC, which is likely to be unavoidably detached that is such a serious issue for subsequent experiments.

The photothermal behavior of FCH was evaluated using an infrared (IR) camera. As shown in Figure 1h, the surface temperature of FCH rapidly increased within the first 5 minutes and stabilized at 33.3 °C, while the side temperature rose to 26.7 °C and remained constant. Under the same conditions, the dry‐state FCH achieved a surface temperature of 63.8 °C and a side temperature of 31.2 °C (Figure 1i). These results demonstrate the rapid photothermal response and excellent heat localization capability of FCH during the light‐to‐heat conversion process.

**Figure 2:**
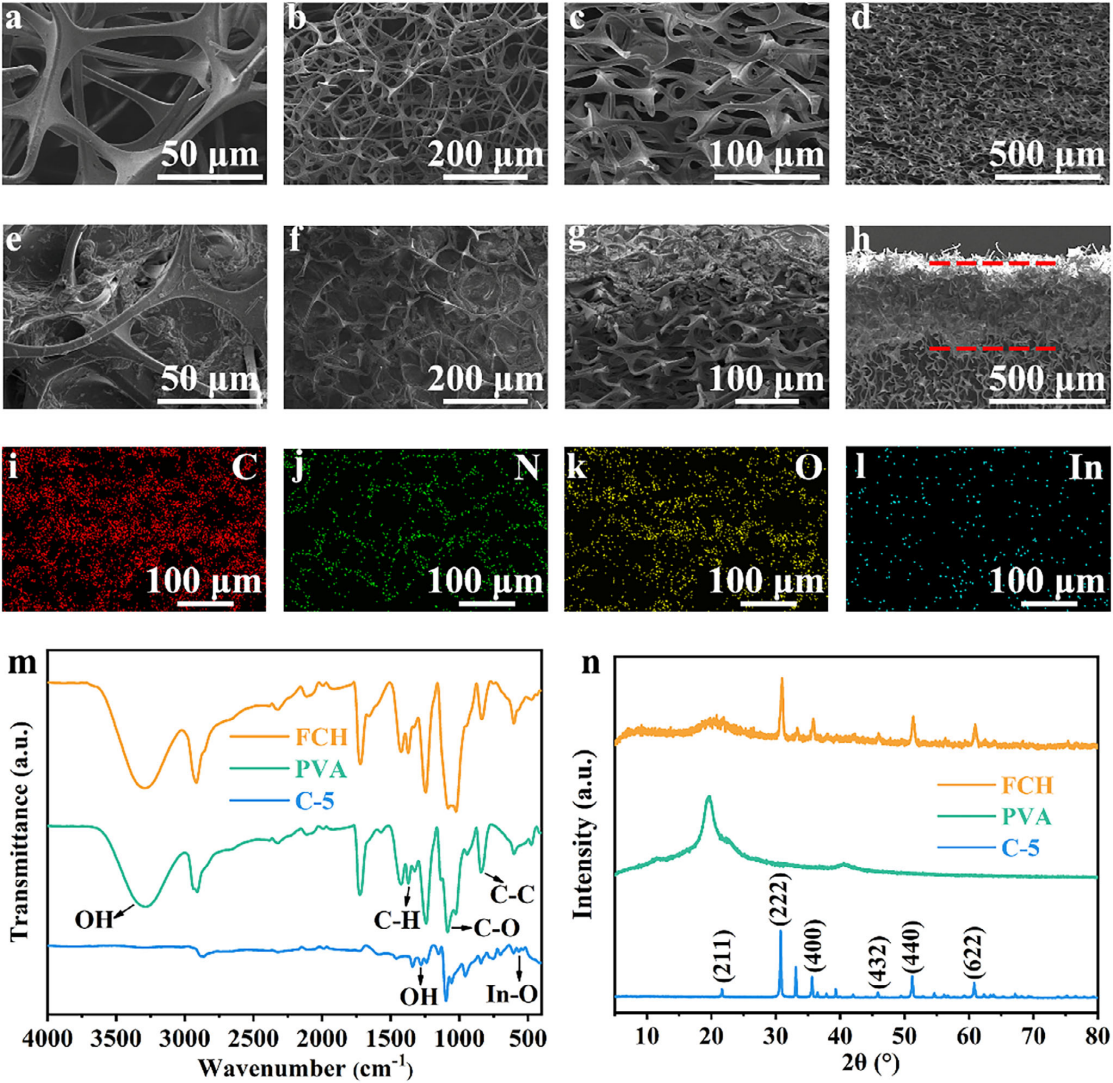
SEM images of a,b) Foam in uncompressed direction, c,d) Foam in compressed direction, e,f) FCH in uncompressed direction, and g,h) FCH in compressed direction at different magnifications. i–l) Element mapping for FCH. m) FT‐IR spectrum and n) X‐ray diffraction patterns of C‐5, PVA, FCH.

To comprehensively showcase the key parameters of the FCH evaporator, detailed characterizations are presented in **Figure** **2**. The surface morphology of the foam substrate is depicted in Figure 2a–d, with Figure 2a,b corresponding to the non‐compressed direction and Figure 2c,d to the compressed direction. Figure 2a,c reveal the interconnected porous channels on the foam surface and interior, while the low‐magnification images in Figure 2b,d demonstrate the dense and porous microstructure of the Foam, which facilitates rapid water absorption and efficient transport. The SEM images of FCH are shown in Figure 2e–h, where Figure 2e,f correspond to the non‐compressed direction and Figure 2g,h to the compressed direction. Figure 2e,f illustrate that the hydrogel thin layer forms interconnected, wrinkle‐like structures. Figure 2g,h further highlight the distinct interface between the hydrogel thin layer and the foam substrate, with the hydrogel thickness measured at ≈ 370 µm. The hydrogel thin layer and foam substrate collaboratively construct a 3D water channel structure within FCH, enabling efficient water transport to the evaporation surface and ensuring a continuous water supply during the evaporation process. The elemental distribution maps (Figure 2i–l) further confirm the uniform distribution of C, N, O, and In elements within the FCH, indicating that the hydrogel thin layer is evenly distributed across the foam substrate without aggregation or stacking.^[^ ^35^ ^]^ These results demonstrate the successful fabrication of FCH evaporators with uniformly distributed hydrogel thin layers.

The TGA spectrum of the photothermal material C‐5 precursor, as shown in Figure S4 (Supporting Information), indicates significant degradation occurring between 394 and 500 °C. Therefore, C‐5 carbonized at 500 °C was selected for subsequent studies. The XPS analysis of the photothermal material, presented in Figure S5 (Supporting Information), further confirms the successful synthesis of C‐5. As shown in Figure 2m, the absorption peaks at 533, 564, and 607 cm^−1^ correspond to the asymmetric stretching vibrations of In‐O bonds,^[^ ^36^ ^]^ indicating the presence of In_2_O_3_ in C‐5. The weak absorption peaks at 1337, 1380, and 1675 cm^−1^ are attributed to ‐OH bending vibrations caused by the absorption of moisture from the air. For pure PVA, a broad ‐OH absorption band is observed in the 3000–3600 cm^−1^ range, while the absorption peaks at 834, 1085, and 1300‐1430 cm^−1^ correspond to the vibrations of C‐C, C═O, and C‐H bonds in the ‐CH_2_ groups of the PVA main chain.^[^ ^37^ ^]^ All these characteristic peaks are present in the spectrum of FCH, confirming the existence of FCH within an interpenetrating network composed of PVA and C‐5, indicating the formation of intermolecular and/or intramolecular interaction‐such as hydrogen bonds or van der Waals forces.

Figure 2n shows distinct diffraction peaks of C‐5 at 2θ = 21.72°, 30.76°, 35.64°, 37.96°, 41.84°, 45.93°, 51.18°, and 60.82°,^[^ ^38^ ^]^ corresponding to the (211), (222), (400), (411), (332), (431), (440), and (622) crystal planes of In_2_O_3_,^[^ ^39^ ^]^ indicating the polycrystalline nature of C‐5. The XRD pattern of pure PVA exhibits a broad diffraction peak near 2θ = 20°, reflecting its semicrystalline nature.^[^ ^37^ ^]^ In the XRD pattern of FCH, the characteristic diffraction peaks of both C‐5 and PVA are observed, further confirming the successful preparation of the hydrogel layer. Figure S6 (Supporting Information) shows the light absorption of C‐5 is 86.22%, which indicates this material has good light absorption ability.

**Figure 3:**
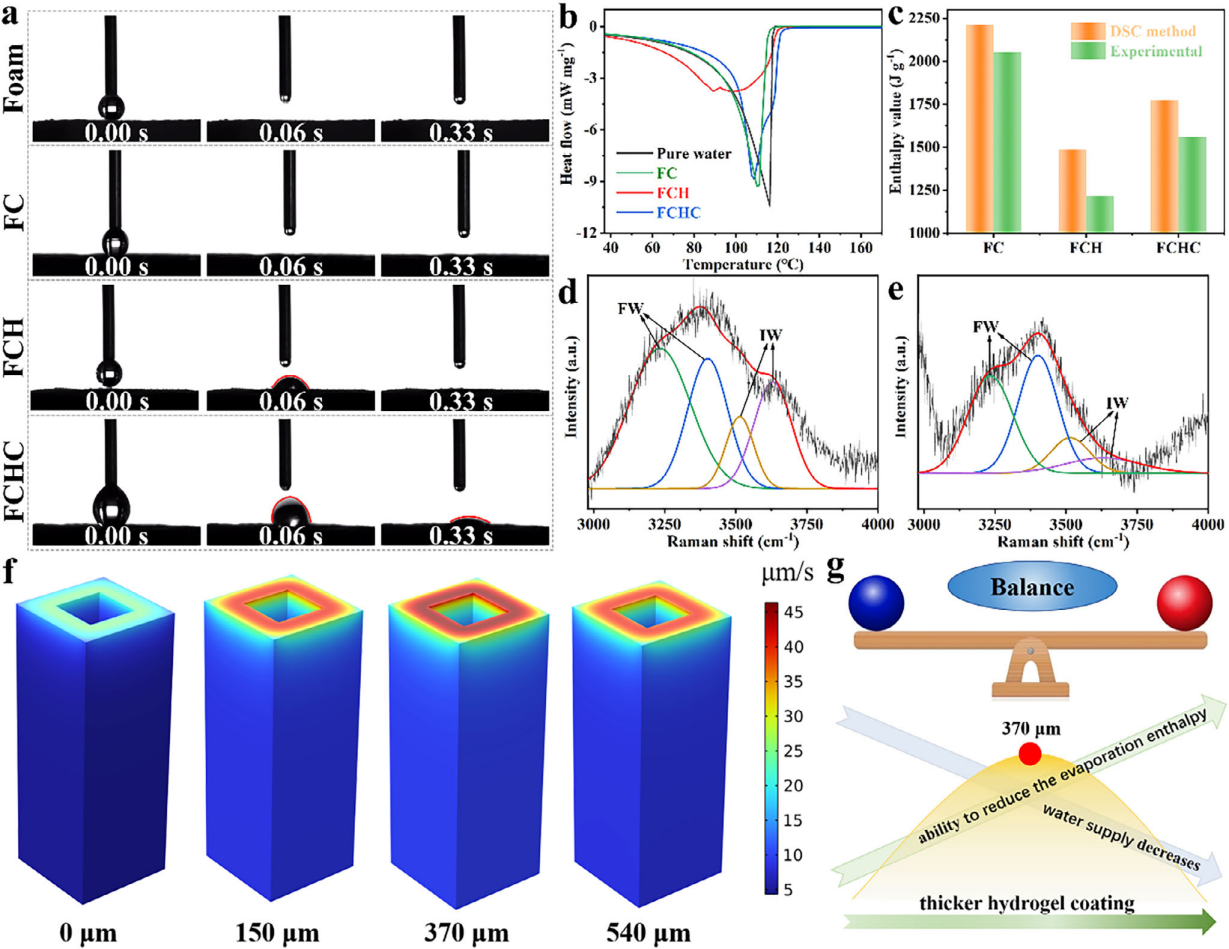
a) Water contact angle test of Foam, FC, FCH, FCHC. b) DSC curves of pure water, FC, FCH, and FCHC. c) Enthalpy measurements of FC, FCH, and FCHC by DSC and experimental methods. d) Raman spectra of FCH and (e) FCHC. f) COMSOL finite element analysis of hydrogel thin layers with different thicknesses. g) Principle of optimal solution of hydrogel thin layer thickness.

To further investigate the interaction between the evaporator and water, **Figure** **3** summarizes the hydrophilic properties of the evaporators and changes in evaporation enthalpy, supplemented by COMSOL simulations to elucidate the underlying mechanism.

As shown in Figure 3a, contact angle measurements reveal that when water droplets come into contact with the surface of Foam or FC, they immediately penetrate into the substrate. For FCH and FCHC, water droplets collapse rapidly upon direct contact with the surface (as indicated by the red lines in Figure 3a; Figure S7, Supporting Information), with the contact line exhibiting pronounced outward spreading behavior. Quantitative results from the contact angle measurements show that the time required for complete wetting, from the initial contact of the droplet to full infiltration, is 0.06 s for both Foam and FC, 0.33 s for FCH, and 0.40 s for FCHC. These findings collectively demonstrate that foam‐based evaporators exhibit superhydrophilicity and excellent wettability. Visual mass transfer experiment confirms rapid water transport from the foam substrate to the hydrogel thin layer (Figure S8, Supporting Information). Upon contact between methyl orange (MO)‐saturated foam and hydrogel‐coated foam (free of photothermal materials to avoid interference), the MO solution diffused into the hydrogel within 0.13 s, advancing ≈ 1500 µm within 0.57 s. This transfer distance exceeds the hydrogel thin layer intrinsic thickness, demonstrating efficient water supply capability for sustained evaporation.

As shown in Figure 3b, the evaporation enthalpy of different evaporators was measured using differential scanning calorimetry (DSC). The results indicate that the evaporation enthalpy of pure water is 2407 J g^−1^, consistent with previously reported values.^[^ ^40^ ^]^ The evaporation enthalpy of FC was 2212 J g^−1^, while FCH exhibited a significant reduction to 1487 J g^−1^, and the distribution of different kind of water can be seen in Figure S9 (Supporting Information). In contrast, FCHC showed a slight increase in evaporation enthalpy to 1775 J g^−1^. This finding fully confirms the previous speculation, demonstrating that only the structure of FCH, which features a unique hydrogel thin layer as the outermost surface, facilitates the formation of intermediate water (IW). IW generation effectively enables water activation, weakens hydrogen bond interactions, and significantly reduces the energy required for water evaporation.^[^ ^41^ ^]^ To better reflect real‐world water evaporation conditions, further tests of evaporation enthalpy were conducted using dark evaporation experiments. As shown in Figure 3c, the evaporation enthalpy values of FC, FCH, and FCHC were 2054, 1215, and 1558 J g^−1^, respectively, consistent with the trend observed in the DSC results but slightly lower in magnitude. This discrepancy arises because DSC measures the enthalpy of a complete dehydration process, whereas actual evaporation tests often involve partial dehydration.^[^ ^42^, ^43^ ^]^

The Raman spectrum of FCH, fitted using Gaussian functions, reveals four distinct peaks (Figure 3d). Peaks corresponding to free water (FW) are located at 3233 and 3401 cm^−1^,^[^ ^17^ ^]^ while peaks associated with IW, characterized by weaker hydrogen bonding, appear at 3514 and 3630 cm^−1^.^[^ ^44^ ^]^ Supplemented by DSC freezing‐melting measurements for verification (Figure S10, Supporting Information). The presence of the hydrogel thin layer activates water molecules, allowing them to preferentially escape from the pores formed by the polymer network in the form of molecular clusters,^[^ ^45^, ^46^ ^]^ thereby significantly reducing the energy required for evaporation. The synergistic interplay between the hydrogel thin layer's nanoscale network and the foam's microscale porosity orchestrates an optimal balance between capillary‐driven water uptake, surface‐assisted retention, and vapor diffusion release. The lower IW/FW ratio for FCHC (0.27) relative to FCH (0.43) (Figure 3e), this reduction may result from the photothermal material loaded on the surface of the hydrogel layer in FCHC, which intensifies the interfacial water restriction effect through strong dipole‐void interactions, thereby increasing the activation energy for water molecule desorption. Under external photothermal irradiation, FW evaporates preferentially, whereas interfacial IW requires overcoming higher energy barrier for desorption due to the anchoring effect. This competitive mechanism ultimately leads to an increase in the apparent evaporation enthalpy. These findings indicate that the hydrogel thin layer must remain as the outermost layer to maximize the reduction of evaporation enthalpy and enhance evaporation performance.

Figure 3f presents the results of the COMSOL finite element analysis. By adjusting the thickness of the hydrogel layer, the evaporation enthalpy and water supply capability of the evaporator were evaluated. The results indicate that the highest water evaporation rate is achieved when the thickness of the hydrogel thin layer is 370 µm; further increasing the thickness leads to a decrease in the evaporation rate. This trend aligns with the experimental results, confirming that there is an optimal thickness for the hydrogel thin layer. Figure 3g further explores the mechanism underlying the optimal thickness of the hydrogel thin layer. The increased thickness of the hydrogel thin layer leads to an increase in IW, which subsequently results in a reduction in evaporation enthalpy (Figure S11 and Table S3, Supporting Information). However, the water supply capability of the hydrogel decreases progressively with increasing thickness (Figure S2, Supporting Information). The interaction between the favorable factor of reduced evaporation enthalpy and the unfavorable factor of diminished water supply ultimately leads to the existence of an optimal hydrogel thickness. At this optimal value, the favorable and unfavorable factors reach a balance, maximizing the water evaporation rate.

### 2.3 Stability Testing of the Evaporator

**Figure 4:**
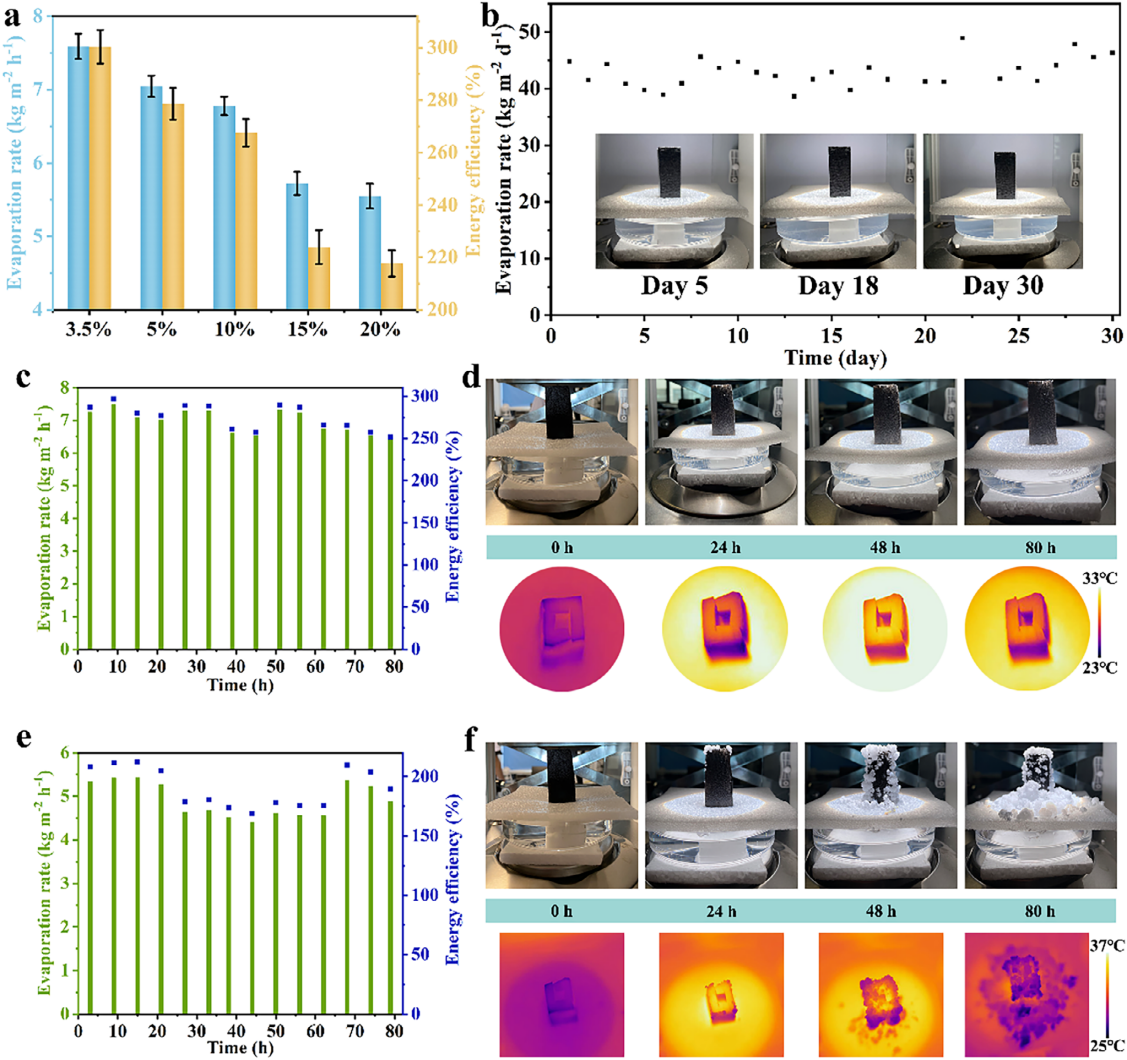
a) Evaporation rate and energy efficiency of FCH with 3.5, 5, 10, 15, 20 wt.% NaCl solution. b) Evaporation rate and physical photographs of FCH in 3.5% NaCl solution for consecutive 30 days. c) Evaporation rate and d) physical and infrared photographs using FCH for 80 h in 3.5 wt.% NaCl solution. e) Evaporation rate and f) physical and infrared photographs using FCH for 80 h in 20 wt.% NaCl solution.

To investigate the applicability of FCH in saline environments, **Figure** **4** presents the evaporation performance of FCH in salt solutions of varying concentrations. As shown in Figure 4a, the evaporation rate of FCH in a 3.5 wt.% NaCl solution is 7.59 ± 0.17 kg m^−2^ h^−1^, which is comparable to its performance in pure water. With increasing salt concentration from 3.5 wt.% to 20 wt.%, the evaporation rate gradually decreases. However, even in a 20 wt.% NaCl solution, the evaporation rate of FCH remains as high as 5.55 ± 0.16 kg m^−2^ h^−1^, demonstrating that the FCH evaporator maintains excellent desalination performance even under high‐salinity conditions.

Figure 4b shows the evaporation performance data of FCH in a 3.5 wt.% salt solution over a continuous 30‐day period. The evaporation rate of FCH remained relatively stable, ranging from 40 to 50 kg m^−2^ d^−1^, and no salt crystallization was observed on the surface of the evaporator throughout the experiment. This indicates that under the alternating day and night conditions typical of natural environments, the advantages of FCH allow it to maintain a long‐term stable evaporation rate and exhibit excellent salt resistance.

Figure 4c,d present the experimental results of FCH evaporation in a 3.5 wt.% salt solution over 80 h under consistent solar irradiation. The data show that the evaporation rate of FCH remained between 6.52 and 7.48 kg m^−2^ h^−1^, and the surface temperature remained relatively constant, with no salt deposition observed. This is likely due to the formation of a concentration gradient within the liquid inside FCH as surface evaporation progresses, which gradually reaches saturation over time.^[^ ^47^ ^]^ When the salt ions in the saturated solution near the evaporation surface flow back into the salt solution through the water transport channels inside FCH, salt crystallization is dissolved, demonstrating the excellent durability of FCH.

The evaporation data of FCH in a 20 wt.% salt solution under continuous illumination for 80 h is shown in Figure 4e,f, with the evaporation rate maintained between 4.51 and 5.43 kg m^−2^ h^−1^. After 24 h of continuous evaporation, salt crystallization was observed at the top edge of FCH, and by 48 h, crystallization extended to the sidewalls of FCH. This phenomenon occurs because, in high‐concentration salt solutions, the salt solution near the evaporation surface gradually reaches saturation, limiting its ability to flow back into the bulk water.^[^ ^48^ ^]^ As evaporation proceeds, salt crystals gradually accumulate. However, after 62 h of continuous illumination, the salt crystals detached automatically under the influence of gravity. Once the crystals fell off, the evaporation rate recovered to 5.37 kg m^−2^ h^−1^, comparable to the performance observed without salt crystallization. This finding demonstrates that FCH evaporators maintain excellent evaporation stability even in high‐concentration salt solutions. This stability is primarily attributed to the superior desalination capability of the hydrogel layer on the FCH, which enables salt particles to detach naturally without significantly affecting the water evaporation process.

**Figure 5:**
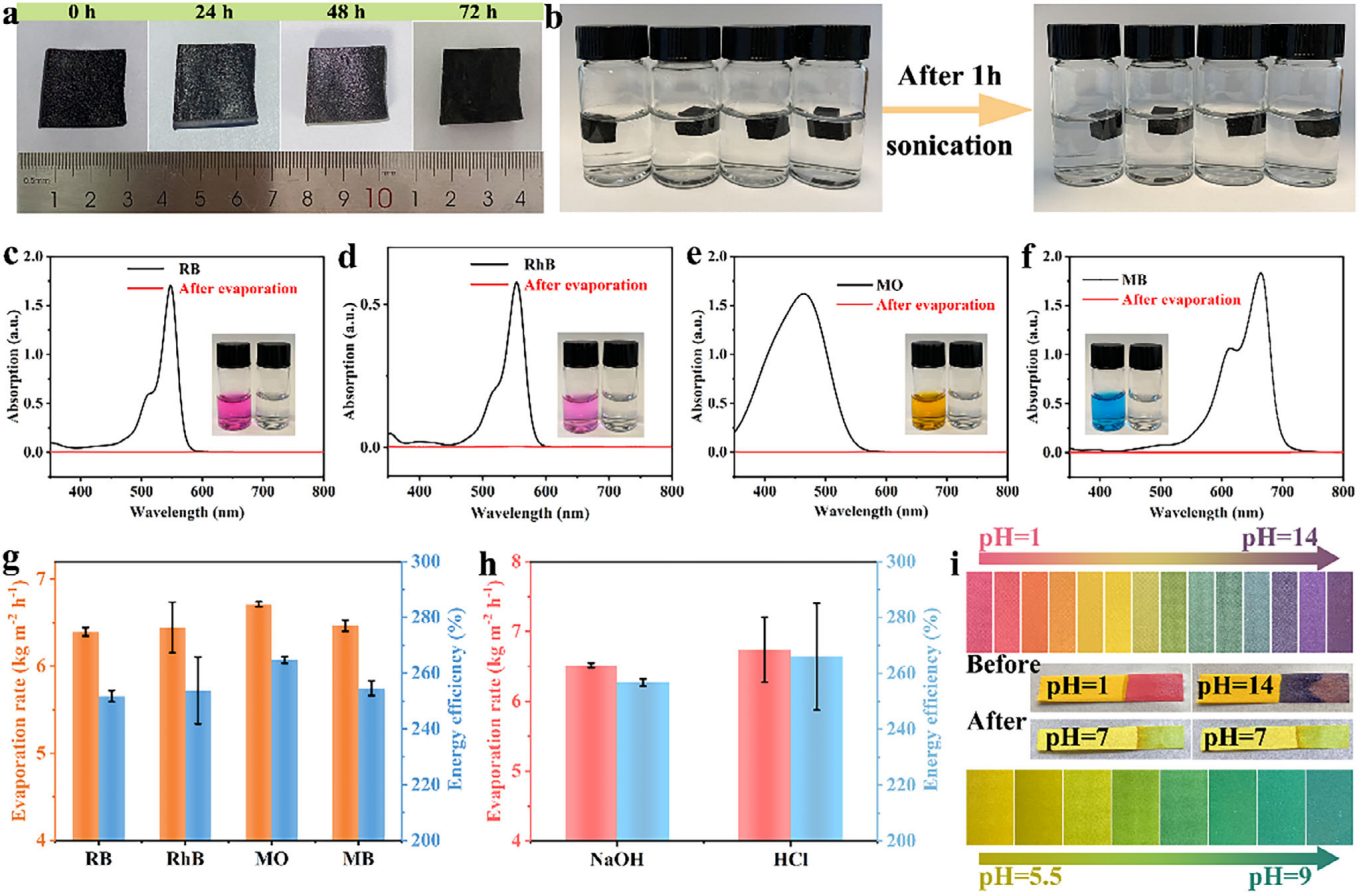
a) FCH changes with time. b) The stability of FCH in pH 1 HCl, pH 13 NaOH, ethanol and acetone after 1 h sonication. Ultraviolet absorption curves of c) RB (bd) RhB e) MO and f) MB before and after evaporation. Evaporation rate and energy efficiency of FCH in g) dye solution h) acid‐alkali solution. i) Comparison of acid and alkali solution before and after evaporation at different pH values.

**Figure** **5** examines the stability of FCH under various environmental conditions. As shown in Figure S14 (Supporting Information), the PVA hydrogel exhibits significant swelling behavior over time, with its volume increasing by ≈25% after 72 h. In contrast, as depicted in Figure 5a, FCH shows no signs of swelling under the same test conditions, demonstrating its exceptional anti‐swelling performance. Additionally, after 5 min of ultrasonic treatment, the FC solution turned black (Figure S15, Supporting Information), indicating significant detachment of the photothermal material. In comparison, FCH retained clear and transparent solutions after 1 hour of ultrasonic treatment in pH 1 HCl solution, pH 1 NaOH solution, ethanol, and acetone, with no observable detachment of the photothermal layer (Figure 5b). These results confirm that the hydrogel thin layer in FCH effectively immobilizes the photothermal material, showcasing its excellent stability and environmental adaptability.

FCH was subsequently utilized for solar‐driven evaporation experiments on organic dye wastewater. Figure 5c–f depict the absorption spectra of wastewater containing methylene blue (MB), methyl orange (MO), Bengal rose red (RB), and rhodamine B (RhB), as well as the treated water samples after evaporation with FCH. The treated water samples appeared clear and transparent, with the characteristic absorption peaks completely eliminated, indicating the complete removal of organic pollutants. Under these conditions, the evaporation rates were 6.39 ± 0.04, 6.44 ± 0.28, 6.71 ± 0.02, and 6.46 ± 0.06 kg m^−2^ h^−1^ (Figure 5g), slightly lower than those observed in pure water systems but still maintaining a relatively high level. These results demonstrate that FCH exhibits excellent evaporation stability and adaptability when treating different types of dye wastewater.

The evaporation performance of FCH was tested under extreme environmental conditions in pH 1 HCl and pH 14 NaOH solutions. The results showed that the evaporation rates in acidic and alkaline solutions were 6.51 ± 0.03 and 6.74 ± 0.46 kg m^−2^ h^−1^, respectively (Figure 5h). As observed from the pH indicator strips in Figure 5i, the water treated by FCH approached neutrality. These findings demonstrate that FCH can maintain high evaporation performance even under challenging extreme conditions while producing clean water. From a scalability perspective, challenges such as consistent material quality, scalable fabrication, durability under variable environments, and environmentally compatible end‐of‐life management should be considered.

### 2.4 Outdoor Testing and Applications

**Figure 6:**
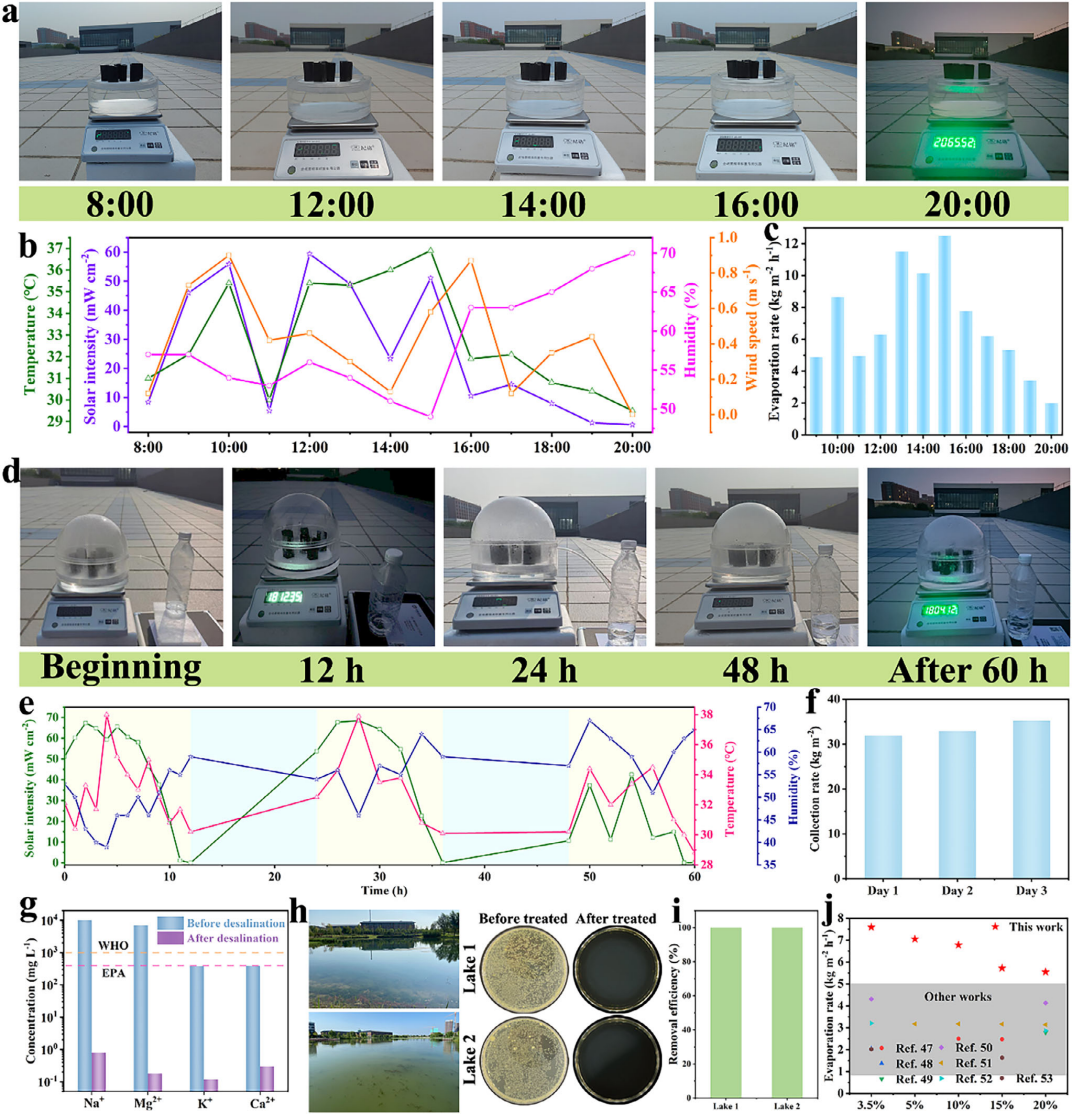
a) Photographs of outdoor experiment at different time. b) Solar intensity, ambient temperature, humidity, and wind speed variation curves and c) evaporation rate at different time. d) Photographs of outdoor collection for three consecutive days. e)Variation curves of ambient temperature, humidity, and natural solar intensity and f) water collection rate in consecutive 3 days of outdoor experiment. g) Concentration of four metal ions before and after desalination of simulated seawater. h) Photographs of bacteria cultivated on agar plates before and after treatment with two lakes. i) Bacterial removal efficiency in two lakes. j) Evaporation performance of the FCH compared with previously reported solar steam generators.

To further evaluate the practical performance of FCH under real‐world conditions, a custom‐made outdoor evaporation (Figure S16, Supporting Information) and water collection system was used for testing. Environmental parameters, including ambient temperature, humidity, wind speed, and solar intensity, were recorded under natural sunlight (**Figure** **6**a,b). The results showed that the actual evaporation rate closely followed the trend of solar intensity, indicating that solar intensity has a significant impact on evaporation performance. Additionally, humidity and wind speed exerted a combined effect on the evaporation rate. As shown in Figure 6c, the maximum evaporation rate of 12.48 kg m^−2^ h^−1^ was observed at 15:00 (solar intensity: 0.51 kW m^−2^, temperature: 36.9 °C, humidity: 49%, wind speed: 0.58 m s^−1^). At this time, high solar intensity, temperature, and wind speed, coupled with low humidity, collectively contributed to achieving the optimal evaporation rate.

During the process of clean water collection, water vapor liquefied at the top of the device, forming droplets that condensed and flowed along the sidewalls into the clean water collection system (Figure S17, Supporting Information). Over the three consecutive experimental days, variations in ambient temperature, humidity, and natural light intensity were recorded (Figure 6d,e). The cumulative water collection amounts of the condensation device were 31.96 , 32.94 , and 35.20 kg m^−2^, respectively, over the three days (Figure 6f). These results demonstrate that the FCH device exhibited relatively stable evaporative clean water collection performance under natural environmental conditions.

The concentrations of Na⁺, Mg^2^⁺, K⁺, and Ca^2^⁺ in simulated seawater before and after purification were analyzed using inductively coupled plasma mass spectrometry (ICP‐MS). The results (Figure 6g) indicate that the ion concentrations after purification were significantly lower than the salinity standards for drinking water set by the World Health Organization (WHO) and the United States Environmental Protection Agency (EPA), with ion removal rates exceeding 99%. These findings demonstrate that the FCH device can efficiently produce clean water from seawater through photothermal interfacial evaporation technology, providing a novel and viable approach to clean water production.

To investigate the potential impact of bacteria and other microorganisms on the evaporation process, lake water was collected from two different lakes in campus as the experimental sample for treatment. As shown in Figure 6h, large number of highly active bacteria were present in the lake water before evaporation treatment. However, after treatment with the FCH device, the bacteria were almost entirely inactivated, achieving a 100% removal rate (Figure 6i). Combined with the purification performance of the FCH device on dye wastewater shown in Figure 5, this demonstrates that the evaporator is capable of efficiently purifying various types of wastewater. The evaporation rate of the FCH in 5% brine (7.04 ± 0.17 kg m^−2^ h^−1^) was slightly lower than that observed in 3.5% brine (7.59 ± 0.17 kg m^−2^ h^−1^), yet it remained significantly higher than the previously reported evaporation rate for solar evaporator (3.17 kg m^−2^ h^−1^).^[^ ^49^ ^]^ Furthermore, a comparison of the evaporation performance of FCH across a series of saline waters (Figure 6j) revealed that it outperformed most solar evaporators reported in previous studies.^[^ ^49^, ^50^, ^51^, ^52^, ^53^, ^54^, ^55^ ^]^

## 3 Conclusion

This study proposes a simple, rapid, and versatile hydrogel thin‐layer modification method, which can significantly enhance the evaporation performance of existing foam‐based evaporators within 40 minutes, achieving an improvement of 15% to 60%. This method is also characterized by its low‐carbon and efficient properties, with a carbon emission of only 0.00462 kg CO_2_ eq kg^−1^. Based on this approach, an FCH evaporator was successfully developed. This device not only exhibits exceptional water supply capacity but also significantly reduces the evaporation enthalpy. Moreover, it effectively addresses the issues of hydrogel swelling in traditional hydrogel evaporators and photothermal material detachment in conventional foam‐based evaporators. Under one‐sun illumination, the FCH evaporator achieved an evaporation rate of 7.93 ± 0.37 kg m^−2^ h^−1^ and demonstrated stable performance in continuous seawater testing for up to 30 days.

In addition, this study combined experimental and theoretical simulations to thoroughly analyze the mechanism of the hydrogel thin‐layer modification method. The results revealed that a hydrogel thin‐layer thickness of 370 µm effectively balances the reduction in evaporation enthalpy with the decline in water supply capacity, thereby maximizing water evaporation performance. This modification method has broad applicability for improving existing foam‐based evaporators and shows excellent potential for practical applications.

## 4 Experimental Section

### Materials

0588 Poly(vinyl alcohol) (PVA) was purchased from Shanghai Chenqi Chemical Technology Co., Ltd. Glutaraldehyde (GA, 25％ in water), N,N‐Dimethylformamide (DMF), Indium(III) nitrate tetrahydrate (InN_3_O_9_), 1,3,5‐benzenetricarboxylic acid (C_18_H_18_O_6_), rose Bengal (RB), methyl Orange (MO), Rhodamine B (RhB), methylene blue (MB) were purchased from Shanghai Macklin Biochemical Co., Ltd. China. Hydrochloric acid (HCl), sodium chloride (NaCl), magnesium chloride (MgCl_2_), dicalcium chloride (KCl), calcium chloride (CaCl_2_), methanol (CH_4_O) was obtained from Chinese Medicine Group Chemical Reagent Co., Ltd. Seaweed foam, polyurethane foam, and melamine foam are purchased from local markets. Deionized water was used throughout the experiments.

### Preparation details—Preparation Method for PVA Hydrogel Thin Layer

Select a suitable foam substrate and wash it multiple times with water and ethanol to remove surface dust and grease. Dry the substrate in an oven at 60 °C. Dissolve 5 g of PVA in 35 mL of water to prepare a 12.5 wt.% PVA solution. Subsequently, add 1.75 mL of 4% HCl solution to the above PVA solution to form the PVA+HCl mixture. Spray 5 mL of the PVA+HCl mixture evenly onto the surface of the foam substrate, followed by spraying 0.25 mL of GA solution. Allow the system to stand for ≈ 40 minutes to enable cross‐linking. The assembled structure forms the foam‐based evaporator with a PVA hydrogel thin layer.

### Preparation Details—Preparation of C5

The precursor of the C‐5 material was synthesized using a hydrothermal method. First, 3 g of trimesic acid was dissolved in 96 mL of DMF to prepare solution A, while 0.6 g of In(NO_3_)_3_ was dissolved in 24 mL of methanol to prepare solution B. Solution A was then added to solution B, and the mixture was stirred for 30 minutes until fully dissolved. The resulting mixture was transferred to a reaction autoclave and heated at 120 °C for 24 h to obtain a precipitate. The precipitate was washed with methanol and centrifuged, and the resulting sample was vacuum‐dried for future use. The dried sample was placed in a tubular furnace under a nitrogen atmosphere, heated to 500 °C at a rate of 5 °C min^−1^, and held for 2 h before cooling to room temperature to obtain the target material C‐5. Similarly, C‐4, C‐6, and C‐7 were prepared by carbonizing the precursor at 400, 600, and 700 °C, respectively.

### Preparation Details—Preparation of Solar Evaporators

A piece of melamine foam, 10× compressed to dimensions of 8 cm (length) × 4 cm (width) × 0.5 cm (thickness), was used as the substrate. The foam was washed multiple times with water and ethanol to remove surface dust and grease and then dried in an oven at 60 °C. The uncoated foam served as the Foam evaporator.

For the preparation of the FC evaporator, a C‐5 aqueous solution (0.1 g mL^−1^) was sprayed onto both the inner and outer surfaces of the foam substrate using a spray gun. For the FCH evaporator, 0.5 g of C‐5 was dispersed in 5 mL of the PVA+HCl mixture solution to prepare solution C. Solution C and 0.25 mL of GA solution were sequentially sprayed onto the inner and outer surfaces of the foam substrate to form a hydrogel thin layer. FCH‐1, FCH‐3, and FCH‐5 denote hydrogel thin layer thicknesses of 150, 370, and 540 µm, respectively.

Finally, to prepare the FCHC evaporator, an additional layer of 0.1 g mL^−1^ C‐5 dispersion was sprayed onto the inner and outer surfaces of the FCH evaporator.

### Solar‐Driven Steam Generation Experiments

The experimental setup for solar‐driven steam generation is illustrated in Figure S18 (Supporting Information). A xenon lamp (CEL‐S500‐T5, Beijing China Education Au‐light Co., Ltd.) was used as the incident light source, providing a solar intensity of 1 kW m^−2^. The light intensity was monitored using a solar power meter (CEL‐NP2000, Beijing China Education Au‐light Co., Ltd.), and the surface temperature of the samples was measured using an infrared thermal imager (FOTRIC 628C, FOTRIC Inc.). Under ambient conditions (28 °C, ≈50% humidity), the evaporators were placed on an analytical balance connected to a computer (measurement precision: 0.0001 g) to record real‐time mass changes, and the evaporation area is defined as the projected geometric surface area of the evaporator top exposed to the light source (viewed from above). The water evaporation performance of the evaporators under simulated sunlight was evaluated, with each sample tested five times to obtain an average. Figure S19 (Supporting Information) shows that the water transport height rapidly reached 4 cm upon contact with the sponge, which was then set as the evaporator height. Under one sun illumination, the energy efficiency was calculated using the following formula:

(1) $$\eta =\frac{\dot{m}h_{LV}}{C_{opt}P_{0}}$$

where $\dot{m}$ is the stable net evaporation rate at ambient temperature, h_LV_ is the total enthalpy of vaporization of water at the corresponding temperature, C_opt_ is the optical concentration, and P_0_ is the standard solar intensity (1 kW m^−2^).

To evaluate the desalination capability of the evaporators in saline water, tests were conducted with solutions of different salinities: 3.5 wt.%, 5 wt.%, 10 wt.%, 15 wt.%, and 20 wt.%. Additionally, solutions of 20 mg L^−1^ methylene blue (MB), methyl orange (MO), rose Bengal (RB), and rhodamine B (RhB) were prepared to assess the evaporators' capacity for removing organic pollutants.

### Solar‐Driven Steam Generation Experiments—Outdoor Evaporation Experiments

Outdoor experiments under natural sunlight were conducted to evaluate the evaporation performance of FCH evaporators under varying environmental factors, such as natural light intensity and temperature. Solar intensity was monitored using a solar power meter (CEL‐NP2000, Beijing China Education Au‐light Co., Ltd.), and the mass loss due to evaporation was measured with an electronic balance (measurement precision: 0.01 g).

### Life Cycle Analysis (LCA)

The scope of the study focuses on different hydrogel preparation processes, including hydrogel foaming and hydrogel freeze‐thaw methods, as well as the hydrogel thin‐layer method mentioned in the study. Functional unit is defined as the carbon emissions generated from the production of hydrogel materials of the same size, which are further used for desalination. This part was committed to calculate the carbon footprint between different technologies.

### Characterization

The surface morphologies of the evaporators were characterized using a field emission scanning electron microscope (SEM, Hitachi Regulus8100, Japan). Fourier transform infrared (FTIR) spectroscopy (Bruker Invenio S, Germany) was employed to scan the infrared spectra of PVA, composite C‐5, and FCH. X‐ray diffraction (XRD) patterns of PVA, composite C‐5, and FCH were measured using an X‐ray diffractometer (Rigaku Smartlab SE, Japan). X‐ray photoelectron spectroscopy (XPS) was conducted to analyze the spectrum of composite C‐5 using a Kratos Axis Supra+ (England). Raman spectra of the evaporators were obtained using a Raman microscope (Renishaw RM2000, England). Contact angles of the evaporators were measured using a contact angle meter (Zhongyikexin SCI3000, China). The enthalpies of the evaporators were determined using a differential scanning calorimeter (Netzsch DSC300, Germany).

## Conflict of Interest

The authors declare no conflict of interest.

## Supporting information



Supporting Information

## Data Availability

The data that support the findings of this study are available in the supplementary material of this article.
