# Supplementary material for: Hydrogel‐Coated Foam Evaporator Achieving Four‐In‐One Benefits for Efficient and Stable Solar‐Driven Water Purification
Source: Adv Sci (Weinh). 2025 Nov 12;13(6):e17280. doi: 10.1002/advs.202517280 (PMC12866709; doi:10.1002/advs.202517280)
Supplement: Supplementary file 1 — Supporting Information [file ADVS-13-e17280-s001.docx]

Emerging strategy: Universal Hydrogel-Coated Foam Evaporator for Sustained High-Efficiency Solar-driven Water Purification

Lele Li^#^, Yanhong Dong^#^, Shu Liu, Xingmin Du, Diandian Yu, Jie Wen,Chenxi Wang, Qian Sun, Bing Liu*, Tao Wu*, Jiahui Yu*

L. Li, Y. Dong, X. Du, D. Yu, J. Wen, Q. Sun, B.Liu, J.Yu

Medical Science and Technology Innovation Center

Shandong First Medical University & Shandong Academy of Medical Sciences

Jinan 250117, China

E-mail: yujiahui@sdfmu.edu.cn;liubing@sdfmu.edu.cn

C. Wang, T. Wu

Department of Chemical and Environmental Engineering

The University of Nottingham Ningbo China

Ningbo 315100, China

E-mail: tao.wu@nottingham.edu.cn

S. Liu

Nottingham Ningbo China Beacons of Excellence Research and Innovation Institute

University of Nottingham Ningbo China

Ningbo 315100, China

Keywords: Solar-driven evaporator, Universal strategy, Sustainable low-carbon technology, Hydrogel-coated foam evaporator

The heat loss by conduction (Q_conduction_) due to contact with body water is calculated as follows:^1^

$Q_{conduction}=cm\Delta T$ (S1)

C is the specific heat capacity of water, m is the mass of the heated water, and ΔT is the change in temperature of the heated water.

Convection heat loss (Q_convection_) due to airflow and heat dissipated to air is calculated as：

$Q_{convection}=Ah\Delta T$ (S2)

A is the area of the evaporator where thermal convection possibly occurs. H is the natural convective heat transfer coefficient (typically 5-10 W m^-2^ K^-1^). ΔT is difference between the surface temperature of an area where thermal convection occurs of the evaporator and the ambient temperature.

The radiative heat loss (Q_radiation_) resulting from the exchange of heat with the surrounding environment can be calculated as follows：

$Q_{radiation}=A\varepsilon\sigma(T^{4}-T_{e}^{4})$ (S3)

A is the area of the evaporator that likely produces thermal radiation, ε is the emissivity of the evaporator, σ is the Stefan-Boltzmann constant (5.669×10^-8^ W m^-2^ K^-4^), T is the surface temperature of the evaporator at steady state, and Te is the ambient temperature adjacent to the evaporator.

The net evaporation rate of the evaporator under light conditions is calculated as：

$\dot{m}=\dot{m_{sun}}-\dot{m_{dark}}$ (S4)

$\dot{m}$ is net evaporation rate, $\dot{m_{sun}}$ is the evaporation rate under sun light, and $\dot{m}_{dark}$ is the evaporation rate under dark conditions

The total enthalpy of water vaporization is expected to include both sensible and latent heat of evaporation. At one atmosphere pressure, the total enthalpy of water vaporization is calculated as:

$h_{LV}=c(T-T_{0})+h_{vap}$ (S5)

T and T_0_ represent the steady evaporation and ambient temperature respectively, and c is the specific heat capacity of water (4.2 J g^-1^ K^-1^). Where the latent heat is calculated as:

$h_{vap}=1.91846\times{10}^{6}\times{(\frac{T}{T-33.91})}^{2}$ (S6)

Meanwhile, calibration corrections for h_vap_ are also experimentally available. Pure water, evaporator, and saturated K_2_CO_3_ solution were simultaneously placed in a closed, dark container and maintained at 25°C and 50% humidity.

$\frac{h_{vap}m}{S}=\frac{h_{vap,water}m_{water}}{S_{water}}$ (S7)

Where S and S_water_ are the evaporator and pure water surfaces respectively, m and m_water_ denote the mass change of the evaporator and pure water respectively after a period of time in a closed dark container, and h_vap,water_ means the latent heat of evaporation of the water.

The light absorption capacity of the series evaporators assessed by UV-Vis-NIR spectroscopy using the following equation:^2^

$\alpha=\frac{\int_{200}^{2500} I(\lambda)(1-R\left( \lambda\right))d\lambda}{\int_{200}^{2500} I(\lambda)d\lambda}\times100\%$ (S8)

The wavelength is λ, I(λ) and R(λ) show the light intensity and reflectance of the solar spectrum at different wavelengths, respectively.

The energy efficiencies for different evaporators is recalculated to incorporate environmental heat exchange, as summarized in Table S1.

Table S1. Comparison of energy efficiency for different evaporators (with and without environmental heat exchange).

| Figure | Name | Energy efficiency (%) | |
| --- | --- | --- | --- |
|  |  | without heat exchange | with heat exchange |
| 1b | FC | 237.75±20.53 | 85.39±3.35 |
|  | FCH | 315.21±15.67 | 98.05±2.56 |
|  | FCHC | 284.69±7.25 | 93.06±1.18 |
| 1c | FCH-1 | 180.64±3.51 | 76.05±0.57 |
|  | FCH-3 | 305.36±8.08 | 96.44±1.32 |
|  | FCH-5 | 241.50±8.20 | 86.00±1.33 |
| 4a | 3.5% | 300.38±6.44 | 95.62±1.05 |
|  | 5% | 278.65±6.00 | 92.06±0.98 |
|  | 10% | 267.58±5.19 | 90.26±0.84 |
|  | 15% | 239.63±6.50 | 83.13±1.06 |
|  | 20% | 217.68±5.04 | 82.10±0.82 |
| 5g | RB | 251.76±2.04 | 87.68±0.33 |
|  | RhB | 253.82±11.97 | 88.01±1.95 |
|  | MO | 264.77±1.16 | 89.80±0.19 |
|  | MB | 254.65±2.62 | 88.14±0.42 |
| 5h | NaOH | 256.72±1.28 | 88.48±2.10 |
|  | HCl | 266.01±19.12 | 90.00±3.12 |
| S1 | SWF | 151.59±3.37 | 71.29±0.55 |
|  | SWF-P | 203.91±11.97 | 79.85±1.95 |
|  | PUF | 193.72±7.34 | 78.18±1.23 |
|  | PUF-P | 224.70±1.23 | 83.25±0.20 |
| S3 | CP | 250.52±20.15 | 87.57±3.29 |
|  | CP+In_2_O_3_ | 206.80±6.53 | 80.32±1.06 |
|  | B-C | 209.42±0.58 | 80.75±0.09 |
|  | B-C+In_2_O_3_ | 178.71±5.09 | 75.73±0.83 |

*Integrated information on life cycle analysis*

Life Cycle Inventory: Consideration of all possible carbon emissions from the production of desalination by the objectives through experimental monitoring and collection of data inventories, as shown in Table S1.

Table S2. Materials and energy input for different hydrogel preparation methods.

|  | Raw materials | Energy |
| --- | --- | --- |
| Foaming method | PVA, HCl, glutaraldehyde, phosphotungstic acid | Mechanical foaming, freeze drying |
| Freeze-thaw method | PVA, HCl, glutaraldehyde, Prulan polysaccharides, Na_2_SO_4_ | Liquid nitrogen, refrigerator |
| **This method** | PVA, HCl, glutaraldehyde | Spray gun |


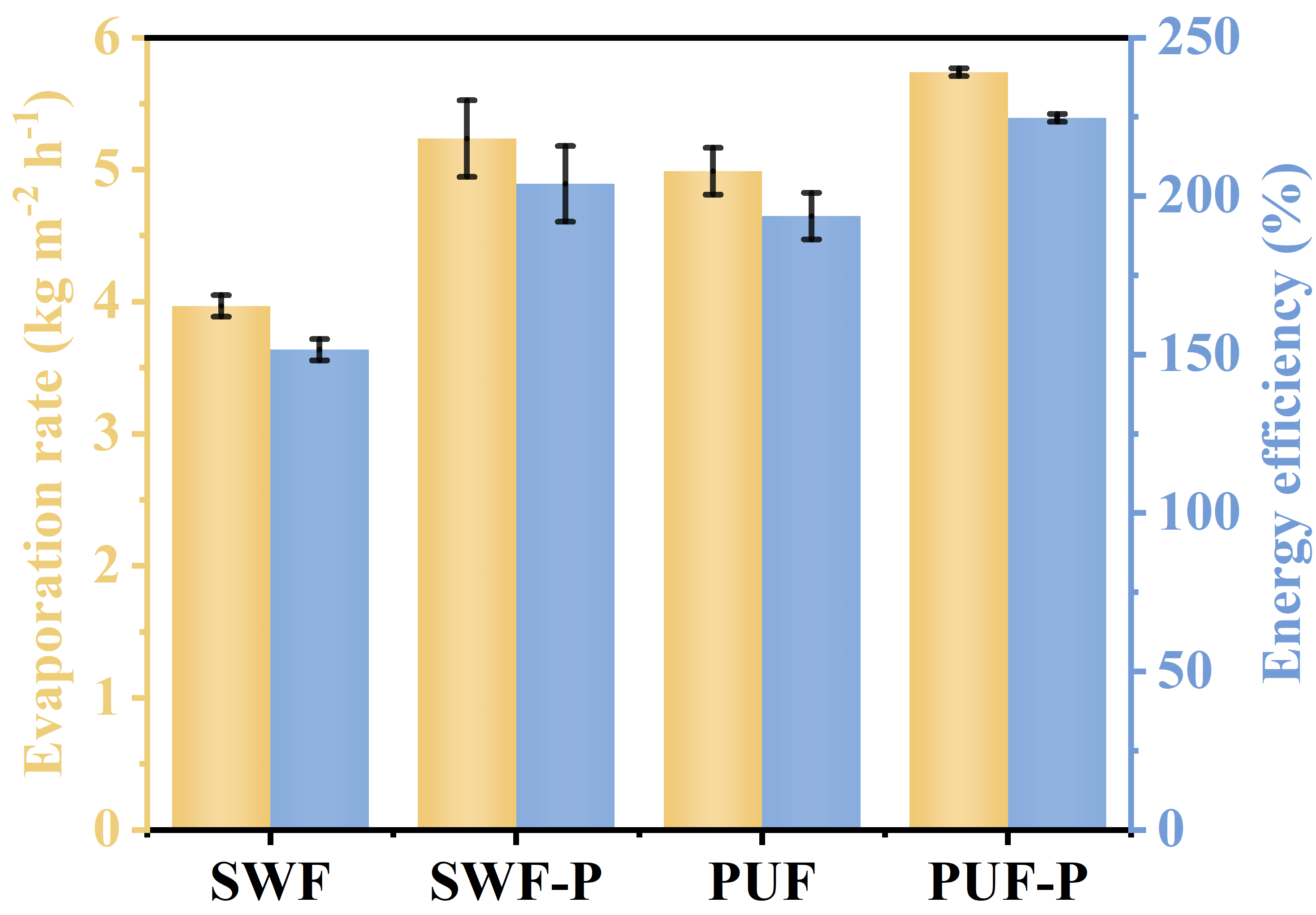


Figure S1. Evaporation rate and energy efficiency of SWF, SWF-P, PUF, PUF-P.

Commercially available seaweed foam (SWF) and polyurethane foam (PUF), which originally showed black color, were selected as the base material. As for the melamine foam, because of its white color, a photothermal material was added to the melamine foam and further prepared as melamine foam-based composite with photothermal material (FC, details in Fig. 1b). Ensure that FC, SWF, and PUF present the same appearance state. The evaporation rates of the SWF, PUF and FC were 3.97±0.08, 4.99±0.17, and 4.90±0.36 kg m⁻² h⁻¹, with energy efficiencies of 151±3.37%, 193%±7.34, and 237±20.53%, respectively. The above three foams modified by hydrogel thin layer were seaweed foam-PVA hydm (SWF-P), polyurethane foam-PVA hydm (PUF-P) and melamine foam-based composite with PVA hydm containing photothermal material (FCH, details in Fig. 1b), with evaporation rates of 5.23±0.28, 5.74±0.03, and 7.93±0.37 kg m⁻² h⁻¹ , and energy efficiencies of 203±11.97%, 224±1.23%, and 315±15.67%, respectively. After modification, the evaporation properties were all improved to different degrees, indicating the method can effectively improve the evaporation performance of different foam-based materials.


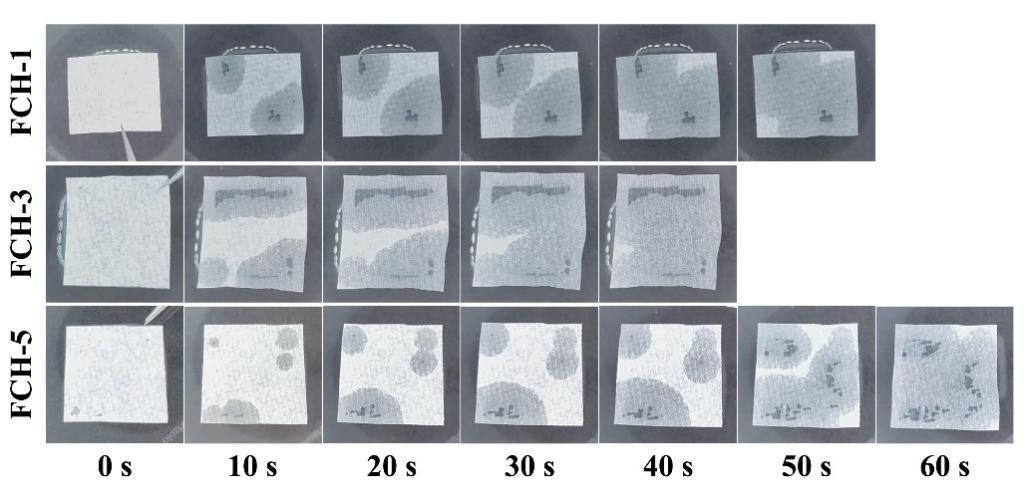


Figure S2. Top surface wetting process of FCH-1, FCH-3, FCH-5.


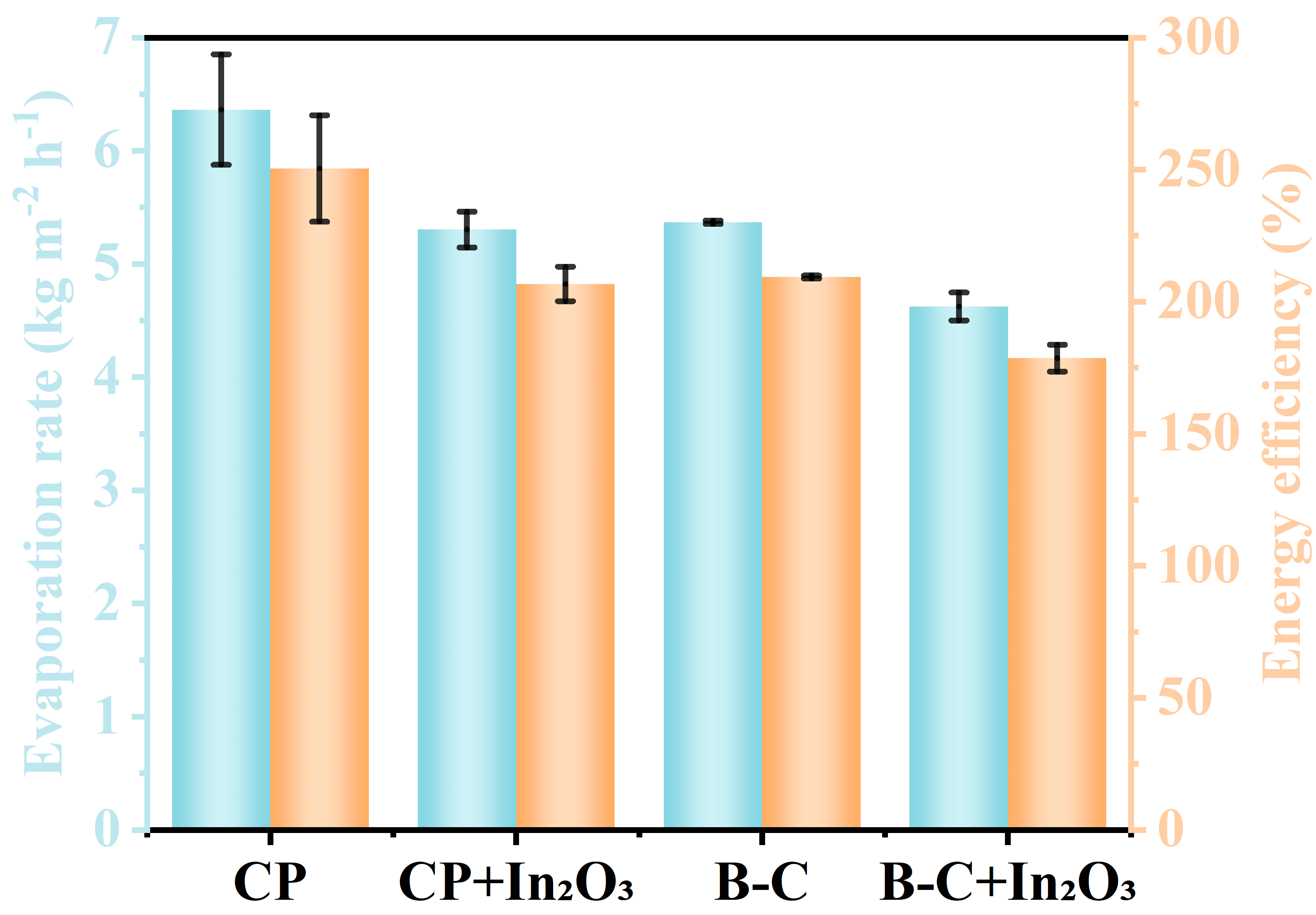


Figure S3. Evaporation rate and energy efficiency of CP, CP+In_2_O_3_, B-C, B-C+In_2_O_3_.

The coating photothermal material (C-5) in the FCH was replaced with carbon powder (CP), carbon powder+In_2_O_3_(CP+In_2_O_3_), 1,3,5-Benzenetricarboxylic acid carbonized at 500 °C for 2 h (B-C), and 1,3,5-Benzenetricarboxylic acid+In_2_O_3_ carbonized at 500 °C for 2 h (B-C+In_2_O_3_), respectively. The evaporation rates of the above evaporators were 6.36±0.48, 5.30±0.15, 5.37±0.01, and 4.62±0.12 kg m^-2^ h^-1^, and the energy efficiencies were 250%±20.15, 206%±6.53, 209%±0.58, and 178%±5.09, which were much lower than that of the FCH evaporator, effectively proving the excellent interfacial evaporation capability of FCH.


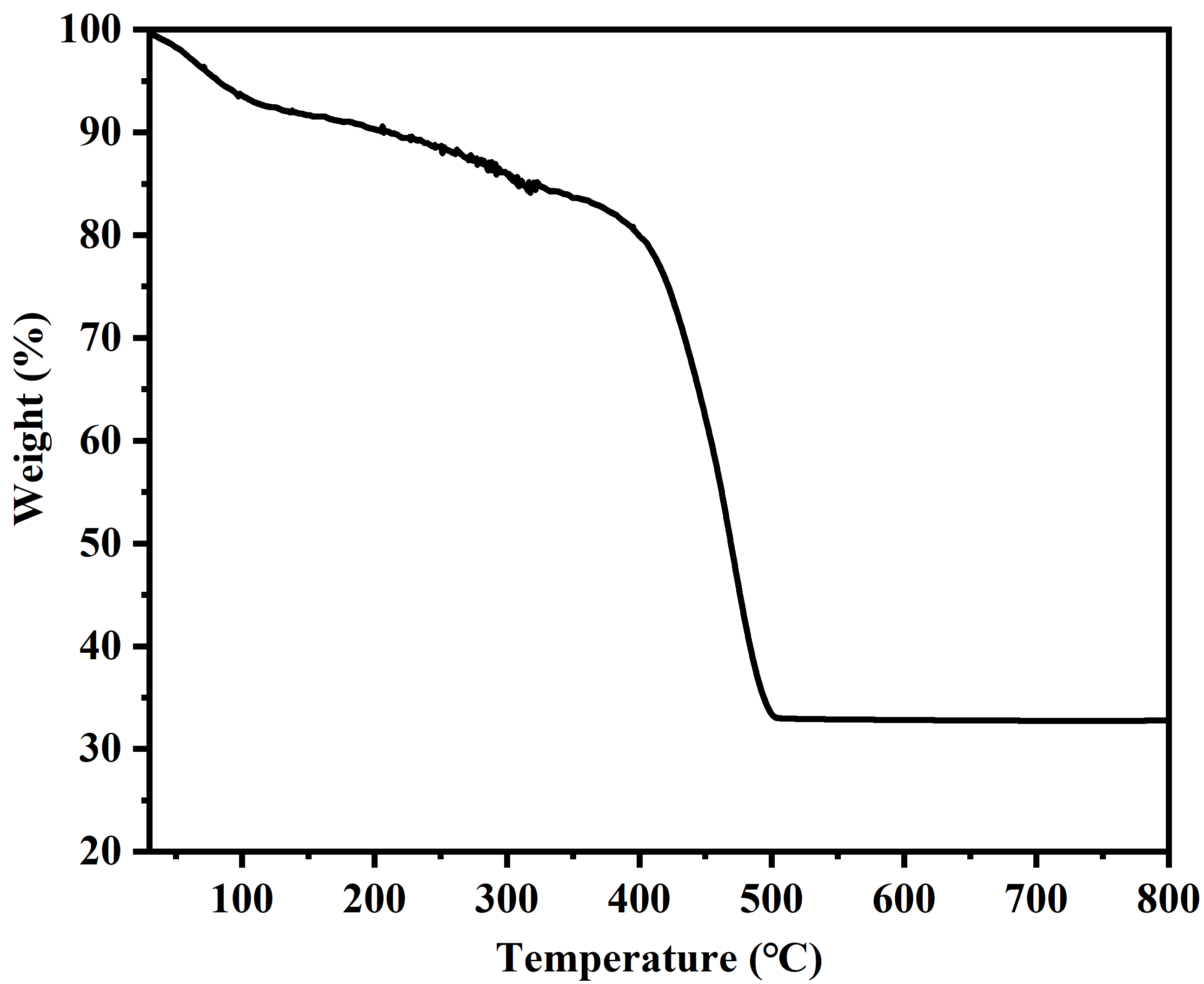


Figure S4. TGA spectra of C-5 precursor.


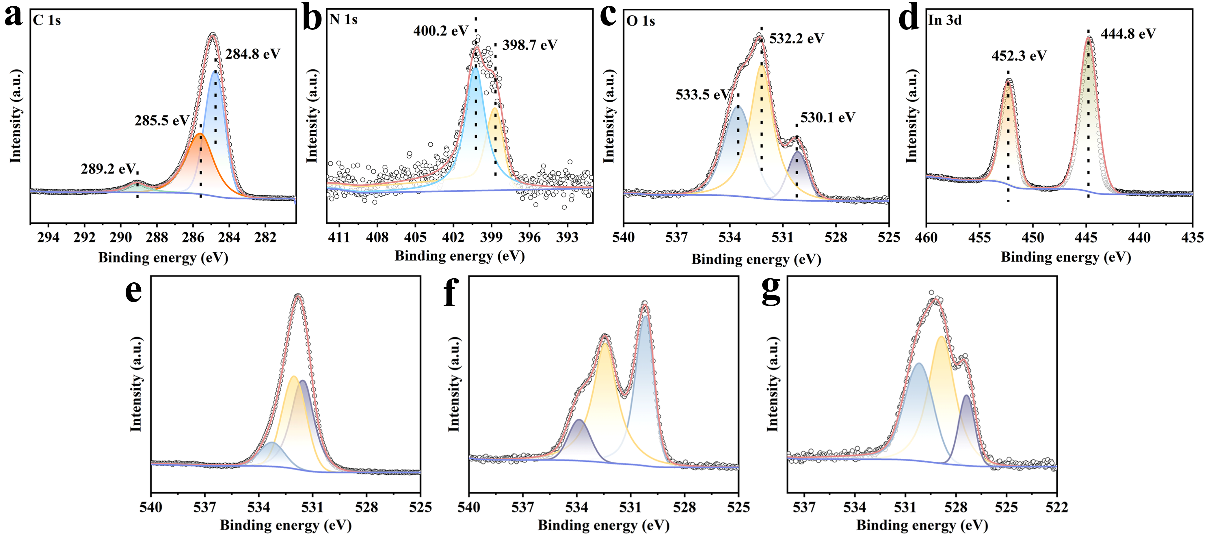


Figure S5. XPS spectra of (a) C 1s (b) N 1s (c) O 1s and (d) In 3d in C-5. XPS spectra of O 1s in (e)C-4, (f)C-6, (g)C-7.

Figure S5(a) shows the C 1s XPS spectrum of C-5, the peak with binding energy at 284.8 eV is attributed to the C-C bond, which is the amorphous carbon on the surface of the material, and the characteristic peaks appearing at 289.2 and 285.5 eV correspond to the O-C=O, C-O bonds.^3^ Figure S5(b) reveals that the binding energies of N 1s are located at 400.2 eV, 398.7 eV, attributed to C-N=C, C-N-H bonds. Figure S5(c) divides the O 1s spectrum into three characteristic peaks at 530.1 eV, 532.2 eV, and 533.5 eV, corresponding to lattice oxygen (O_lattice_), oxygen vacancy (O_vacancy_), and adsorbed oxygen (O_absorbed_), respectively.^4^ According to C_Ovacancy_=A_Ovacancy_/(A_Olattice_+A_Ovacancy_+A_Oabsorbed_), the O_v_ content of C-5 is 50.78%, and the O_v_ content of C-4, C-6, and C-7 are 43.92%、48.58%、47.51%(Figure S5e-g). Figure S5(d) shows that In 3d5/2 and In 3d3/2 have characteristic peaks at 444.8 eV and 452.3 eV, respectively, with an energy difference of 7.5 eV between In 3d5/2 and In 3d3/2, indicating that In is present as In^3+^in C-5.^5^


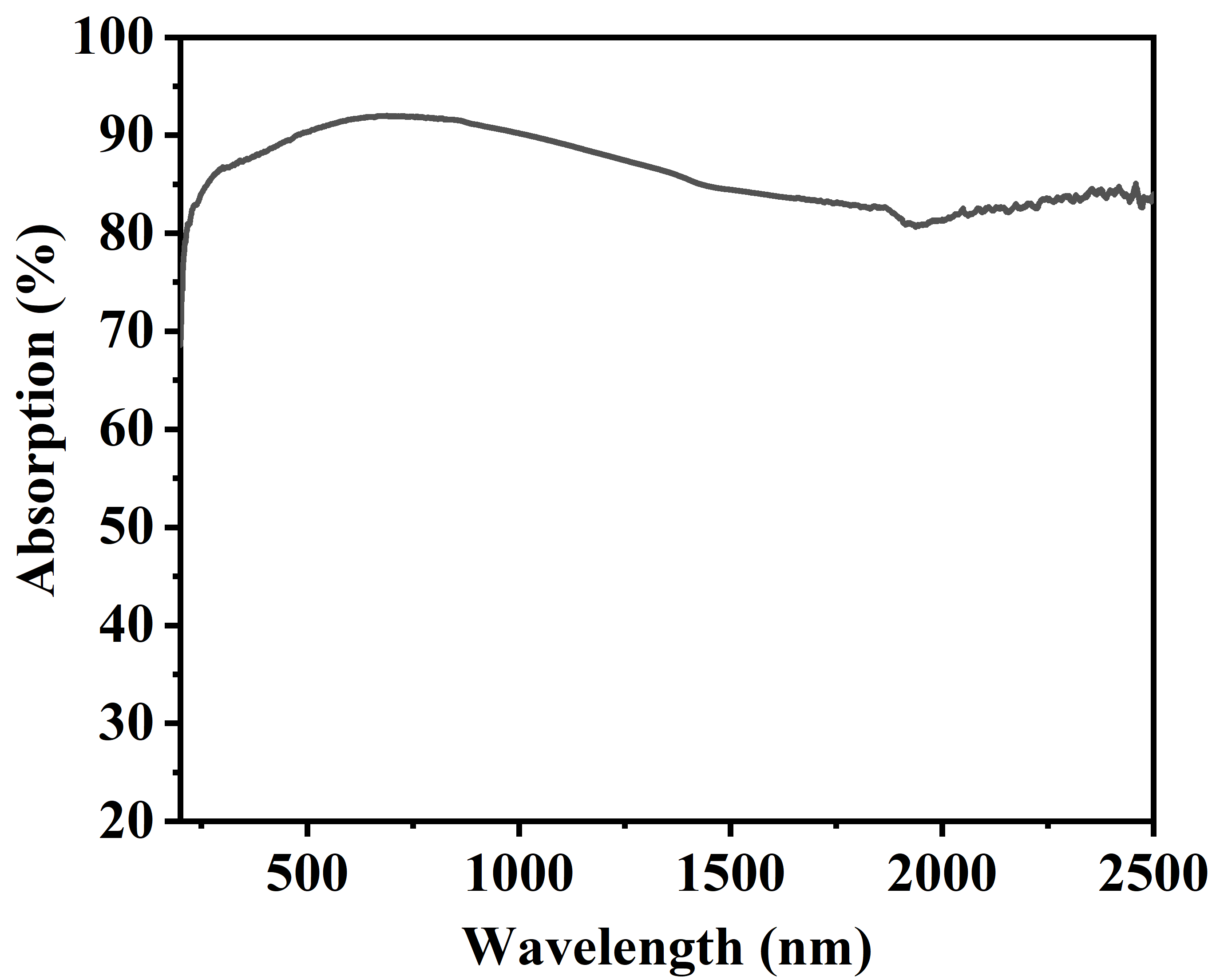


Figure S6. Absorption spectra of C-5.


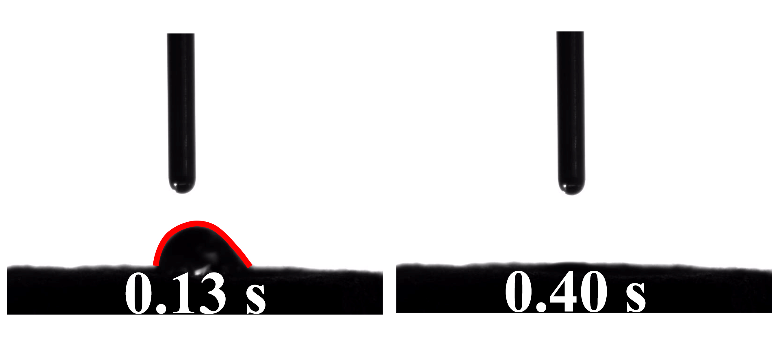


Figure S7. Water contact angle of FCHC.


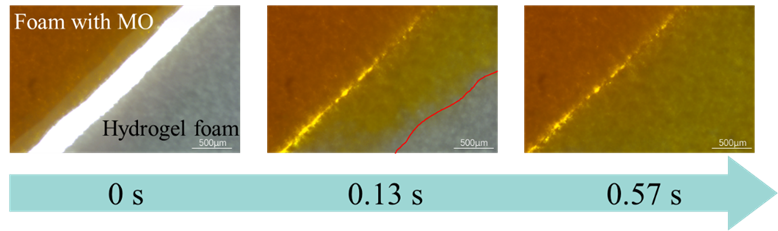


Figure S8. Water transportation from pure foam to hydrogel thin layer foam.


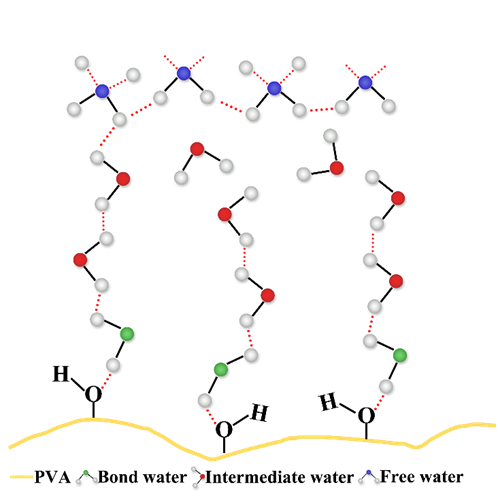


Figure S9. Distribution of BW, IW, and FW in polymer networks.

The hydrophilic groups on the polymer chains form hierarchical hydration structures with water molecules, generating intermediate "bridging water clusters." These clusters disrupt the continuous hydrogen-bond network of water, significantly lowering the apparent evaporation enthalpy and thereby enhancing the evaporation rate under identical energy input.


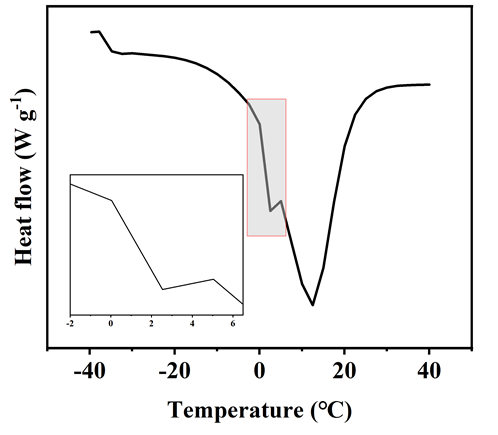


Figure S10. DSC curves of FCH freezing–melting process.

DSC freezing–melting measurements^6^ indicate that the hydrogel thin layer contains freezable bound water (FBW≈IW) with a melting point of approximately 0 °C, alongside (FW) melting at about 2 °C.


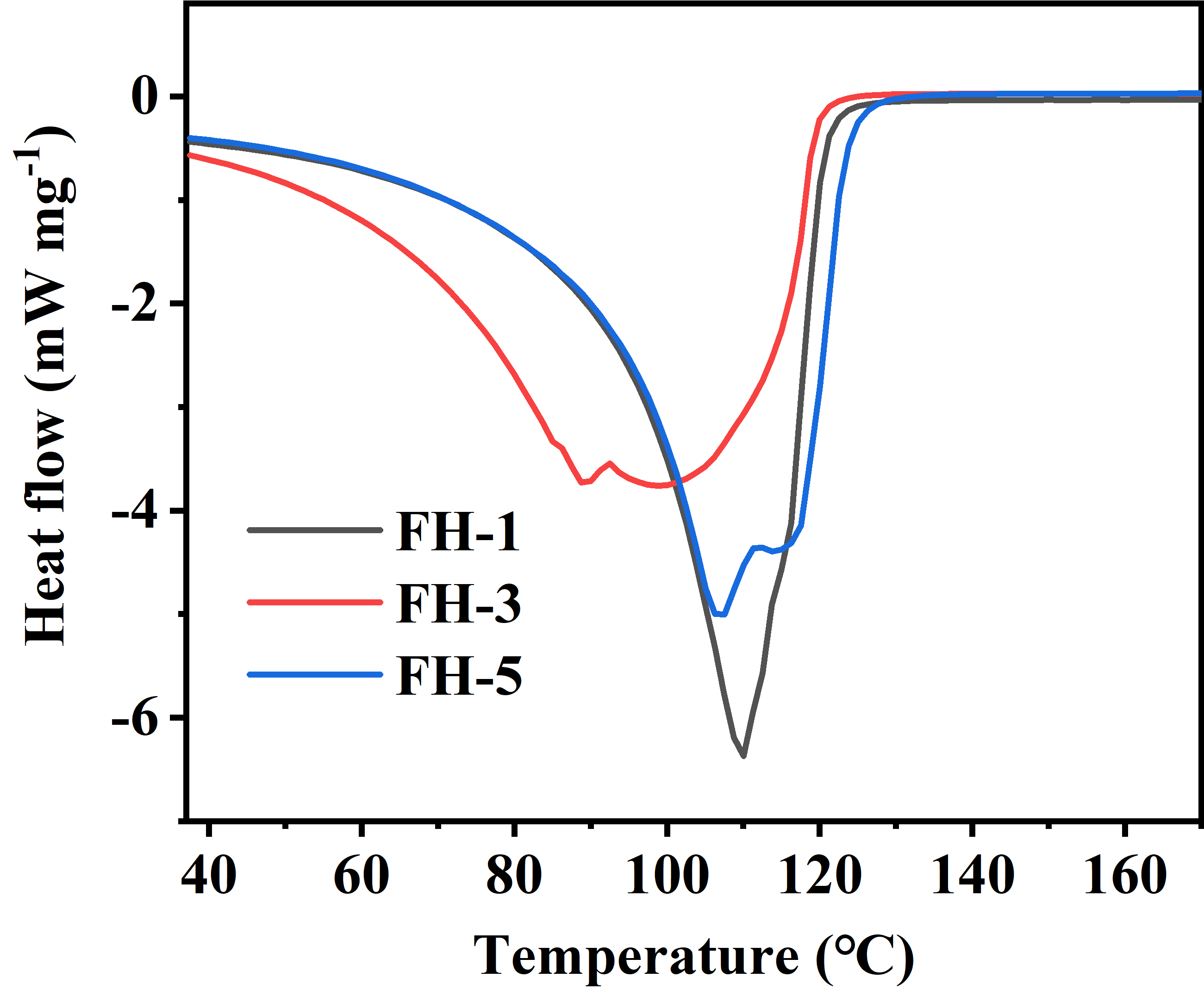


Figure S11. DSC curves of FCH-1, FCH-3, FCH-5.

Table S3. Enthalpy values of FCH-1, FCH-3, FCH-5.

|  | Thickness | Value |
| --- | --- | --- |
| FCH-1 | 150 μm | 1554 J g^-1^ |
| FCH-3 | 370 μm | 1487 J g^-1^ |
| FCH-5 | 540 μm | 1488 J g^-1^ |

Table S4. Physical properties of FCH with the parameter selection for the simulation.

| Name | Expression | Value | Description |
| --- | --- | --- | --- |
| h_foam_ | 80 mm | 0.08 m | Foam height |
| w_foam_ | 30 mm | 0.03 m | Foam width |
| w_hole_ | w_foam/2_ | 0.015 m | Foam pore size |
| w_dom_ | 100 mm | 0.1 m | Width of simulation area |
| h_dom_ | 120 mm | 0.12 m | Height of simulation area |
| eps_por_ | 0.3 | 0.3 | Porosity |
| th_film_ | 0 um | 0 m | Film thickness |
| U_water_ | 1 um s^-1^ | 1e^-6^ m s^-1^ |  |
| rho | 1000 kg m^-3^ | 1000 kg m^-3^ | Water density |
| mu | 1e^-3^ Pa s | 0.001 Pa·s | water viscosity |
| C_basic_ | 5 mm s^-1^ | 0.005 m s^-1^ | Basic evaporation rate |
| deltaH | 6 kcal kg^-1^ | 25104 J kg^-1^ |  |
| rho_steam_ | 2 kg m^-3^ | 2 kg m^-3^ | Steam density |
| I_rad_ | 800 W m^-3^ | 800 W m^-3^ | Solar radiation intensity |

Table S5. Formulas used in COMSOL.

| Name | Expression | Unit |
| --- | --- | --- |
| K_evap_ | $\frac{C\times\left( ht.psat-mt.phi\times ht.psat \right)}{p+1\left[ atm \right]}$ | m s^-1^ |
| C | $\mathrm{derate}\left( {th}_{film}\left[ 1/um \right] \right)\times deltaH\left( {th}_{film}\left[ 1/um \right] \right)\times C_{basic}$ | m s^-1^ |
| Q_evap_ | $-\mathrm{deltaH}\times{rho}_{steam}\times K_{evap}$ | W m^-2^ |


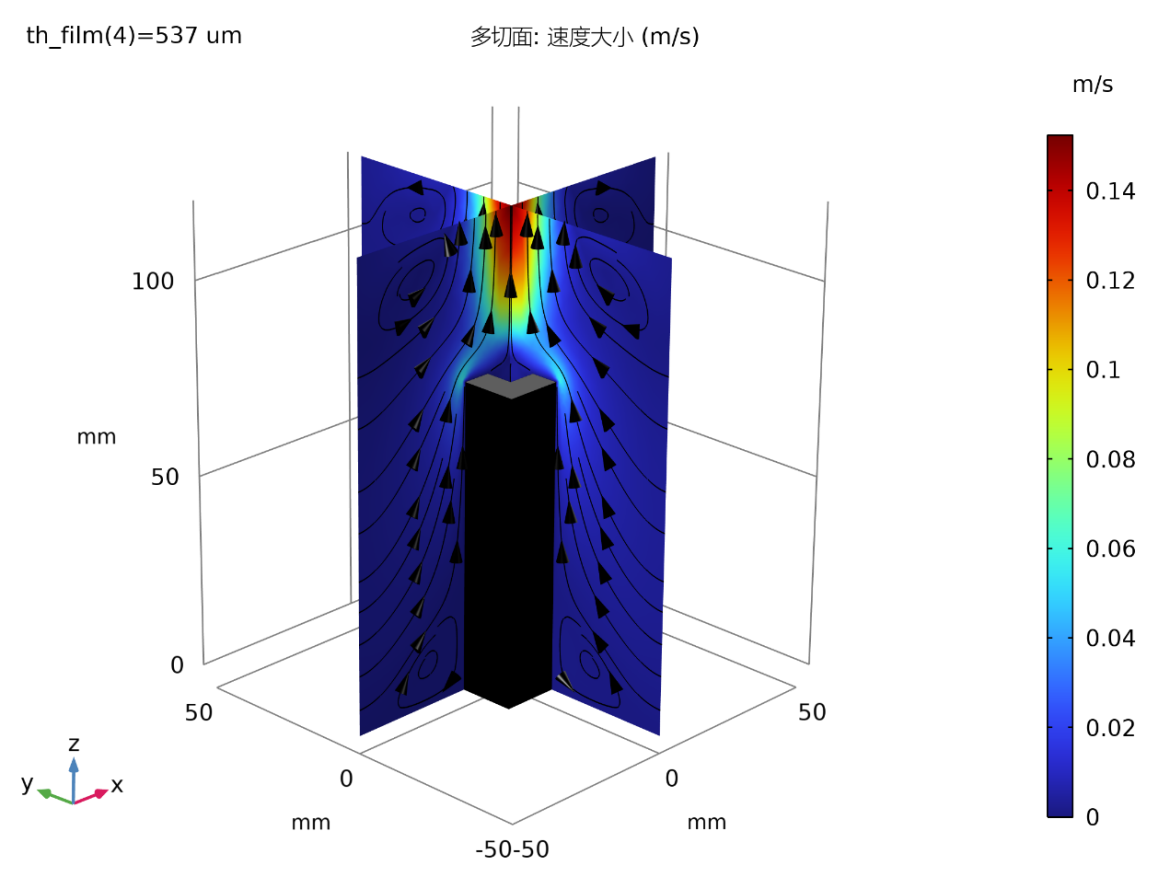


Figure S12. Distribution of escape velocity of vapor.

Under natural conditions, water vapor tends to flow vertically upwards, showing convergence towards the middle. It is worth noting that the vapor escape velocity directly above the FCH can be as high as 0.14 m s^-1^.


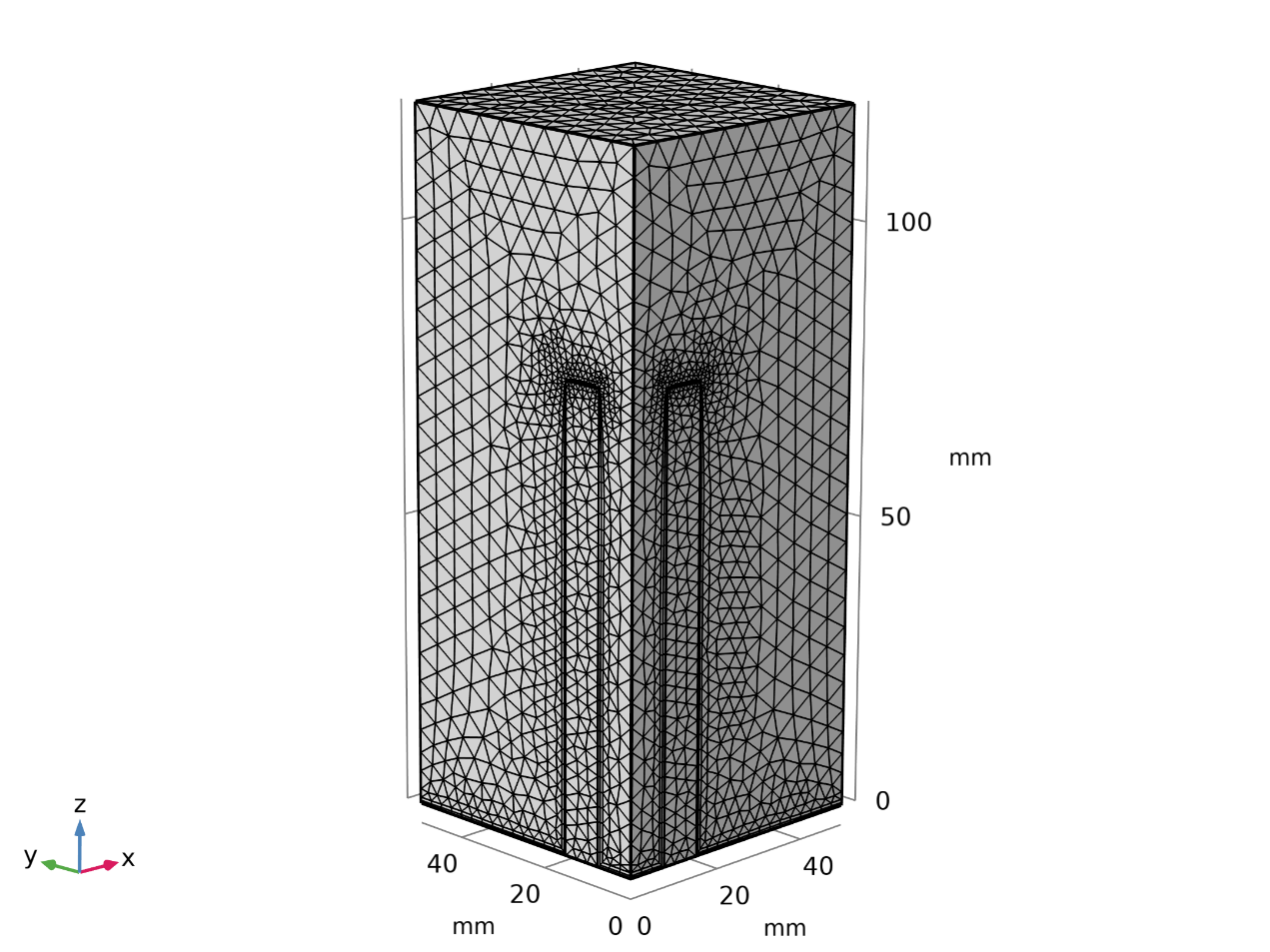


Figure S13. The meshing discretization of FCH in finite element method.


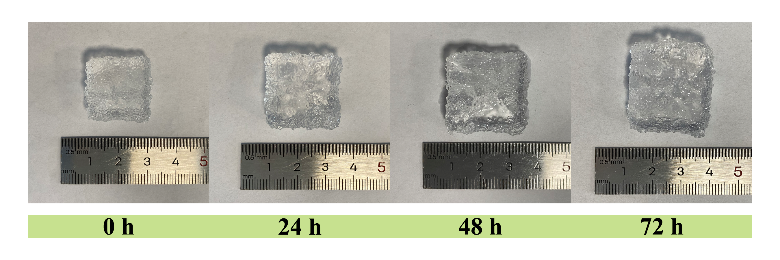


Figure S14. The hydrogel swelling process.


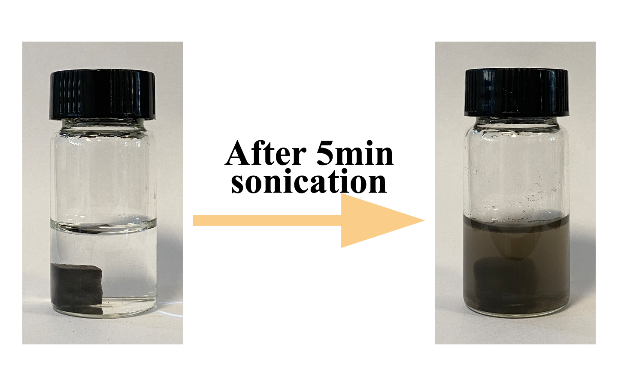


Figure S15. The stability of FC in pure water after 5min sonication.


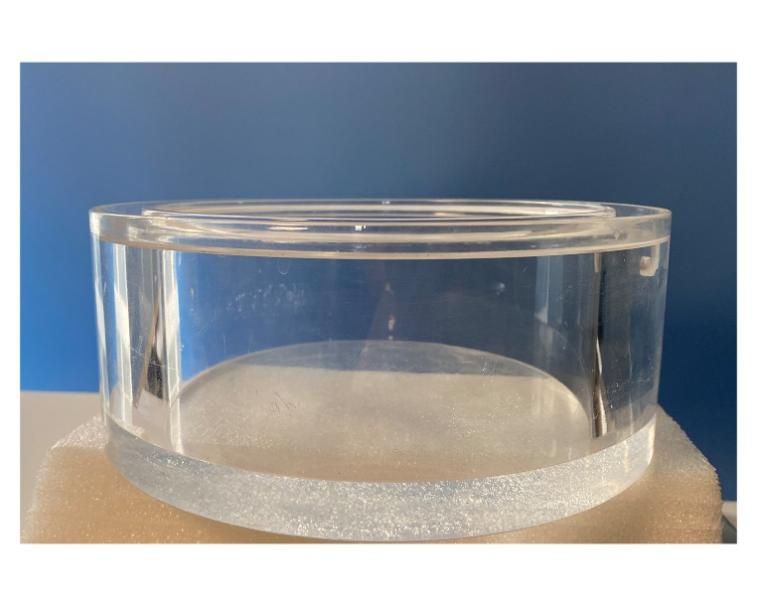


Figure S16. Outdoor evaporation units.


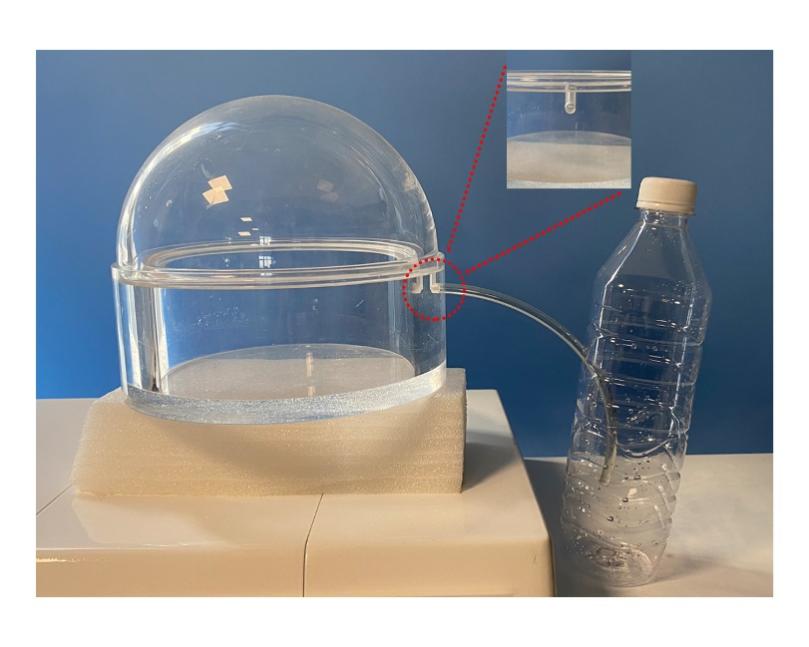


Figure S17. Outdoor evaporation collection units.


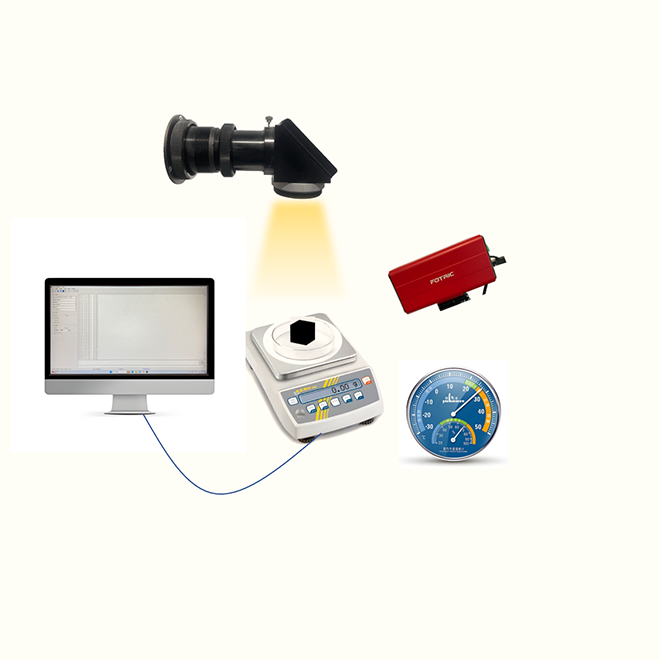


Figure S18. The simulated evaporation system in this work.


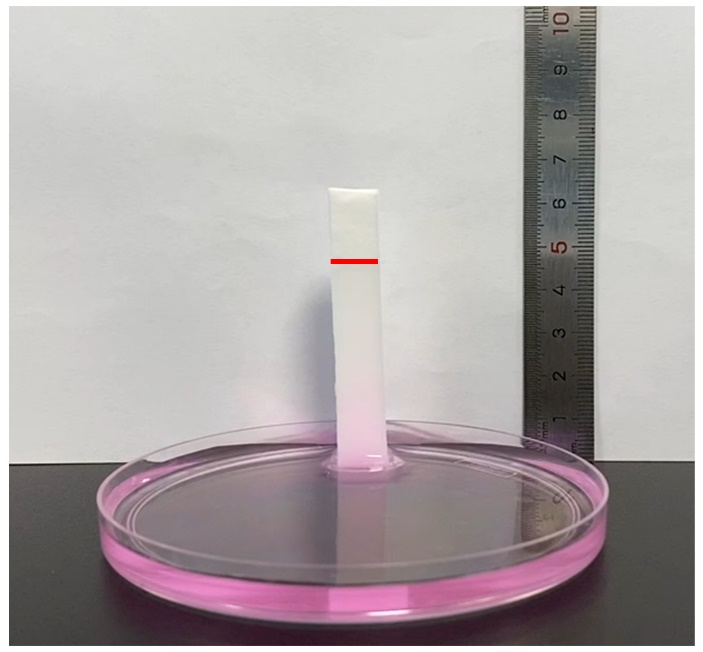


Figure S19. Water transfer height test for pure foam.

REFERENCES

1. Xiong, X.; Arshad, N.; Tao, J.; Alwadie, N.; Liu, G.; Lin, L.; Yousaf Shah, M. A. K.; Irshad, M. S.; Qian, J.; Wang, X., Hierarchical Ti3C2/BiVO4 microcapsules for enhanced solar-driven water evaporation and photocatalytic H2 evolution. *Journal of Colloid and Interface Science* **2024,** *668*, 385-398.

2. Lim, H. W.; Park, S. H.; Lee, S. J., 3D thermoresponsive hydrogel with enhanced water uptake and active evaporation for effective interfacial solar steam generation. *Desalination* **2023,** *550*, 116368.

3. Cheng, Q.; Huang, M.; Xiao, L.; Mou, S.; Zhao, X.; Xie, Y.; Jiang, G.; Jiang, X.; Dong, F., Unraveling the Influence of Oxygen Vacancy Concentration on Electrocatalytic CO2 Reduction to Formate over Indium Oxide Catalysts. *ACS Catalysis* **2023,** *13* (6), 4021-4029.

4. Luo, L.; Fu, L.; Liu, H.; Xu, Y.; Xing, J.; Chang, C.-R.; Yang, D.-Y.; Tang, J., Synergy of Pd atoms and oxygen vacancies on In2O3 for methane conversion under visible light. *Nat. Commun.* **2022,** *13* (1).

5. Shi, Y.; Su, W.; Wei, X.; Song, X.; Bai, Y.; Wang, J.; Lv, P.; Yu, G., Highly active MIL-68(In)-derived In2O3 hollow tubes catalysts to boost CO2 hydrogenation to methanol. *Fuel* **2023,** *334*, 126811.

6. Chen, Y.; Duan, J.; Yu, J.; Ye, D.; Zhan, Y.; Jiang, X., Cost-effective and salt-resistant spongy hydrogel with strengthened Hofmeister effect for accelerated solar-driven evaporation in brine. *Desalination* **2025,** *616*, 119341.
